# Genetic diversity, structure, and effective population size of an endangered, endemic hoary bat, ʻōpeʻapeʻa, across the Hawaiian Islands

**DOI:** 10.7717/peerj.14365

**Published:** 2023-01-25

**Authors:** Corinna A. Pinzari, M. Renee Bellinger, Donald Price, Frank J. Bonaccorso

**Affiliations:** 1 Tropical Conservation Biology and Environmental Science Graduate Program, University of Hawaiʻi at Hilo, Hilo, Hawaiʻi, United States of America; 2 Hawaiʻi Cooperative Studies Unit, University of Hawaiʻi at Hilo, Hawaiʻi National Park, Hawaiʻi, United States of America; 3 Pacific Island Ecosystems Research Center, U.S. Geological Survey, Hawaiʻi National Park, Hawaiʻi, United States of America; 4 School of Life Sciences, University of Nevada - Las Vegas, Las Vegas, NV, United States of America

**Keywords:** Hawaiian hoary bat, Hawaiʻi, Population genetics, Endangered species, Island bat conservation, *Lasiurus*, Mitochondrial DNA, Microsatellites, Bats

## Abstract

Island bat species are disproportionately at risk of extinction, and Hawaiʻi’s only native terrestrial land mammal, the Hawaiian hoary bat (*Lasiurus semotus)* locally known as ʻōpeʻapeʻa, is no exception. To effectively manage this bat species with an archipelago-wide distribution, it is important to determine the population size on each island and connectivity between islands. We used 18 nuclear microsatellite loci and one mitochondrial gene from 339 individuals collected from 1988–2020 to evaluate genetic diversity, population structure and estimate effective population size on the Islands of Hawaiʻi, Maui, Oʻahu, and Kauaʻi. Genetic differentiation occurred between Hawaiʻi and Maui, both of which were differentiated from Oʻahu and Kauaʻi. The population on Maui presents the greatest per-island genetic diversity, consistent with their hypothesized status as the original founding population. A signature of isolation by distance was detected between islands, with contemporary migration analyses indicating limited gene flow in recent generations, and male-biased sex dispersal within Maui. Historical and long-term estimates of genetic effective population sizes were generally larger than contemporary estimates, although estimates of contemporary genetic effective population size lacked upper bounds in confidence intervals for Hawaiʻi and Kauaʻi. Contemporary genetic effective population sizes were smaller on Oʻahu and Maui. We also detected evidence of past bottlenecks on all islands with the exception of Hawaiʻi. Our study provides population-level estimates for the genetic diversity and geographic structure of ‘ōpeʻapeʻa, that could be used by agencies tasked with wildlife conservation in Hawaiʻi.

## Introduction

Island bat species are disproportionately at risk of extinction due to both natural and human disturbances to their ecosystems, with half of all threatened bat species recorded as island endemics, and almost half of those species found on single islands only (Jones et al., 2009; Fleming & Racey, 2009). The Hawaiian Islands are home to a single extant native bat, the Hawaiian hoary bat (*Lasiurus semotus*), or ʻōpeʻapeʻa in the Hawaiian language (Tomich, 1986; Gomes, 2020). Hawaiian island biota epitomize the struggle of threatened species, with hundreds of terrestrial endemic species facing declines in the Anthropocene due to resource degradation, changes in climate and habitat, competition and predation from invasive species, overharvest, loss of key pollinators, and disease (Cox & Elmqvist, 2000; Dobson et al., 1997; Fortini et al., 2013; Bellard et al., 2017; Paxton et al., 2018). Despite the term “Anthropocene” being conceptually used by both scientists and non-scientists to describe the present epoch in which anthropogenic activities are having a significant effect on the global environment, it currently has no formal status in the Divisions of Geologic Time (US Geological Survey, 2018). However, bat populations are currently in decline globally, and conserving future bat diversity may rely on understanding anthropogenic drivers of decline and exploring mitigation measures (Voigt & Kingston, 2016).

The ʻōpeʻapeʻa is listed as endangered by State and Federal agencies due to the lack of available information on abundance, distribution, critical habitat needs and population size (US Fish and Wildlife Service, 1998; Endangered Species Recovery Committee & State of Hawaii Department of Land and Natural Resources Division of Forestry and Wildlife, 2021). Current potential threats include timber harvest practices during the pupping season, entanglement on barbed-wire fencing, bioaccumulation from pesticides, and fatal collisions with wind turbines and vehicles (US Fish and Wildlife Service, 2011; US Fish and Wildlife Service, 2021). Conservation and recovery of endangered bat populations are most challenging when little is known about ecological requirements, population size and trends, and genetic diversity, such as is the case for the ʻōpeʻapeʻa.

Insights provided by flight modeling and phylogenetic studies suggest ʻōpeʻapeʻa became established in Hawaiʻi consequent to trans-Pacific flights from the coast of North America by northern hoary bats, *Lasiurus cinereus,* over one million years ago, while divergence between islands is estimated to have begun half a million years ago (Bonaccorso & McGuire, 2013; Baird et al., 2015; Russell et al., 2015; Baird et al., 2017; Pinzari et al., 2020). The northern hoary bat and ʻōpeʻapeʻa are sister species that are highly mobile, insectivorous, and foliage roosting; however, demographic isolation over millennia has led to notable phenotypic differences. These differences include variations in flight and echolocation characteristics, foraging behavior, pelage coloration, and reduction in body size in ʻōpeʻapeʻa (Jacobs, 1993; Jacobs, 1996; Barclay, Fullard & Jacobs, 1999; O’Farrell, Corben & Gannon, 2000).

Molecular population genetic studies using mitochondrial and nuclear gene sequences have recognized two distinct mitochondrial clades of bats present in the Hawaiian Islands. One clade is present solely within Hawaiʻi, and a second is present in Hawaiʻi and across North America (Baird et al., 2017; Russell et al., 2015). Baird et al. (2017) suggested that the two clades in Hawaiʻi represent distinct species of ʻōpeʻapeʻa, with limited hybridization; however, a recent genome-wide study by Pinzari et al. (2020) examined 110k single nucleotide polymorphisms in 23 ʻōpeʻapeʻa from four Hawaiian Islands, including individuals from both mitochondrial clades, and found geographic segregation of bat populations by island but no evidence to support the presence of two distinct species. Furthermore, no published studies demonstrate morphological or acoustic characteristics that support species level distinction within ʻōpeʻapeʻa mitotypes. Here we follow the taxonomic nomenclature of Wilson et al. (2019), Pinzari et al. (2020), and Simmons & Cirranello (2022), in which all Hawaiian bats are recognized as a single species, *Lasiurus semotus*, and refer to all bats within the Hawaiian Islands as ʻōpeʻapeʻa. For an alternative taxonomy proposed for Hawaiian bats, see Baird et al. (2015) and Baird et al. (2017).

Detecting population size, trends, and dispersal characteristics using traditional methods such as direct counts or mark-recapture in solitary, mobile, cryptic tree roosting bats such as ʻōpeʻapeʻa is extremely challenging (O’Shea, Bogan & Ellison, 2003; Schorr, Ellison & Lukacs, 2014). Although a single acoustic study based on detectability and occupancy indicated a slightly positive contemporary population for Hawaiʻi Island, no estimates of population size are available because the number of bats cannot be enumerated using acoustic methods (Gorresen et al., 2013). Recent exploratory population viability assessments conducted for ʻōpeʻapeʻa found that outcomes under various take levels were highly sensitive to initial population sizes and that larger initial census sizes (>5,000 bats) were necessary to avoid population declines over the next 50 years (Endangered Species Recovery Committee & State of Hawaii Department of Land and Natural Resources Division of Forestry and Wildlife, 2021). Although contemporary acoustic surveys do show ʻōpeʻapeʻa to be widely distributed on all high islands in the archipelago (Montoya-Aiona et al., 2020), virtually nothing is known about bat dispersal within or between islands. It has been suggested that seasonal aggregations of bats (<22 individuals) in coastal areas between August and September may give rise to interisland migrants (Kepler & Scott, 1990). Prior genetic studies having limited sample sizes have focused on colonization history and ancestral divergence of ʻōpeʻapeʻa from continental lineages but have not addressed island-wide population structure or gene flow within the Hawaiian Islands (Pinzari et al., 2020).

Given the limitations of field techniques, genetic approaches offer alternative means to provide estimates of population structuring, gene flow between and within islands, and infer genetic population size for ʻōpeʻapeʻa (Luikart et al., 2010; Moussy et al., 2013; Dool, 2020). The use of both mitochondrial and microsatellite markers allows assessment of historical and contemporary population structure and demographics that may be useful in conservation efforts for island bat species (Dool et al., 2016; Flanders et al., 2009; Salgueiro et al., 2007; Wright et al., 2018). For example, maternally inherited mitochondrial DNA (mtDNA) can provide estimates of female site fidelity and dispersal, while polymorphic nuclear microsatellite DNA can provide information for both sexes (Vonhof & Russell, 2015). Genetic techniques are available for monitoring the persistence and resilience of populations, and effective population size estimates show promising results in monitoring long term population trends in bats (Schwartz, Luikart & Waples, 2007; Wright, Schofield & Mathews, 2021). Additionally, microsatellites have been used to describe the population structure, effective size, and cryptic genetic diversity for tree roosting bats including the endangered Florida bonneted bat (*Eumops floridanus*) (Austin et al., 2022), as well as several species of lasurine bats in North America (Korstian, Hale & Williams, 2015; Pylant et al., 2016; Chipps et al., 2020).

No microsatellite marker studies of ʻōpeʻapeʻa have been published, despite this marker class remaining an important tool for landscape population genetic questions and suitability in cases where the use of SNPs are not a satisfactory tool (Wang, 2010; Hauser, Athrey & Leberg, 2021). Our present study builds upon prior research, utilizing ʻōpeʻapeʻa samples collected over more than a decade, while applying standard conservation genetic approaches to test hypotheses related to panmixia and island connectivity history of this bat species. In this study we use 18 nuclear microsatellite markers for hoary bats, and a region of the mtDNA cytochrome c oxidase I (COI), as well as two genes on the sex chromosomes to explore the following hypotheses: (1) ʻōpeʻapeʻa populations on Hawaiʻi, Maui, Oʻahu, and Kauaʻi Islands show genetic structure, (2) contemporary gene flow and dispersal by ʻōpeʻapeʻa between these islands is restricted, (3) contemporary genetic effective population sizes of ʻōpeʻapeʻa are smaller than historical effective population sizes, and (4) dispersal is primarily male driven with philopatry for roosting sites exhibited by females. The resulting information on population boundaries, baseline genetic diversity, and effective population size provides data and insights that may be used for management actions targeting future recovery of this island bat.

## Materials & Methods

### Tissue sampling

**Figure 1:**
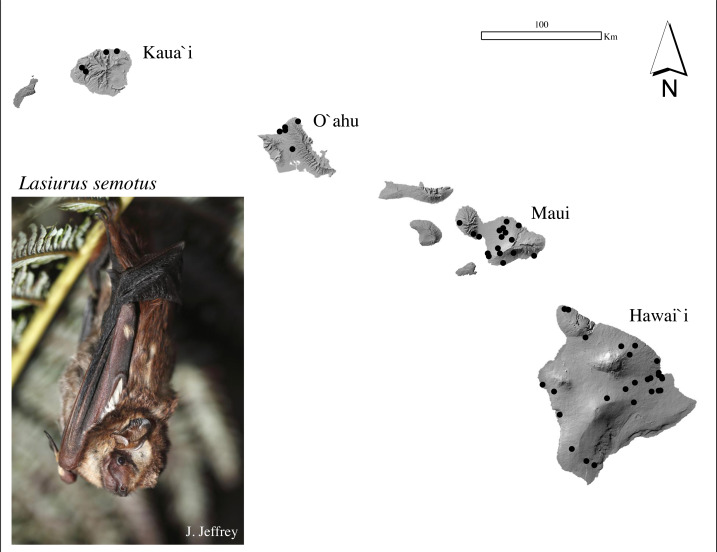
Sample collection map and photograph of a Hawaiian hoary bat, locally known as ‘ōpe‘apea. Generalized locations of ʻōpeʻapeʻa tissue samples used in mitochondrial and microsatellite analysis collected between 1988 and 2020 across four Hawaiian Islands. Photograph Credit: Jack Jeffrey.

Previous genetic studies of ʻōpeʻapeʻa consisted of small sample sizes with limited geographic coverage on each island (Baird et al., 2015; Russell et al., 2015; Baird et al., 2017; Pinzari et al., 2020). In order to provide robust analyses capable of addressing population genetics questions, population genetic data from a wider variety of resources was necessary. We assembled a biological sample collection using wing membrane and/or muscle sourced from 339 individuals across four Hawaiian Islands (Fig. 1, Table S1). The mtDNA analyses included 321 individuals (180 males; 141 females) and microsatellite analyses included 298 individuals (176 males; 119 females; three unknown). A total of 280 bats were examined for both mtDNA and microsatellite DNA. Tissues were obtained between 2005 and 2020 from live bats (*n* = 197) captured in mist-nets according to Kunz & Parsons (2009), and from carcasses (*n* = 142, 2007–2020) submitted from wind energy facilities, the Hawaiʻi State Division of Forestry and Wildlife, the U.S. Geological Survey’s National Wildlife Health Center Honolulu Field Station, the Hawaiʻi Wildlife Center (2007–2020), and from four museum skins (accessions: BPBM177155, BPBM178067, BPBM184307, BPBM184308) during 1988–1999, vouchered in the Bernice Pauahi Bishop Museum (Honolulu, Hawaiʻi). Carcasses were refrigerated or frozen upon discovery. Muscle tissues from necropsies were stored at −20 °C. A sterile 3-mm biopsy punch was used to obtain tissue from each wing of carcasses and live bats. Wing tissues were stored in NaCl-saturated 20% DMSO or on silica gel desiccant beads in the field, then frozen at −20 °C until extraction. USGS procedures are consistent with the guidelines for capture, handling, and care of mammals according to the Institutional Animal Care and Use Committee (IACUC) through the University of Hawaiʻi (#04-039-17) and the American Society of Mammalogists (Sikes, Care & Mammalogists, 2016). Biological samples from bats were collected under Federal and State of Hawaiʻi endangered species collection permits: USFWS TE003483; DLNR-DOFAW WL05-03 through WL19-52.

### Molecular methods

DNA was extracted from wing and muscle tissue using a DNeasy Blood and Tissue Kit (Qiagen, Valencia, CA, USA) following the manufacturer’s protocol for purification of total DNA from animal tissues, or a QIAmp DNA Micro Kit (Qiagen, Valencia, CA, USA) with protocol modifications for wing punches (Corthals et al., 2015). Isolated DNA was amplified for 1 mtDNA marker, 20 nuclear microsatellite loci, and two genes on the X and Y sex chromosomes.

To assess population structure, female-biased gene flow, and maternal effective population size, a 657-bp region of the mtDNA gene cytochrome c oxidase I (COI) was amplified using the forward primer HCO2198 (5′-TAAACTTCAGGGTGACCAAAAAATCA-3′) and the reverse primer LCOI490 (5′-GGTCAACAAATCATAAAGATATTG-3′) (Folmer et al., 1994). Polymerase chain reaction (PCR) was conducted using Illustra Hot Start mix PCR beads (GE Healthcare, Chicago, IL, USA) in 25-µL reaction volumes, containing 20.5 µL of sterile water, 0.5 µL of each primer (10 µM), and 2.5 µL of genomic DNA template. PCR cycling conditions followed Hebert et al. (2003). PCR products were cleaned using Exo-Sap (Affymetrix, Santa Clara, CA, USA). Sanger sequencing of PCR products using forward and reverse primers was performed *via* ABI Prism 3500 Genetic Analyzer (Applied Biosystems, Carlsbad, CA, USA) at the University of Hawaiʻi at Hilo Core Genomics Facility (UHH CGF) or ABI 3730xl DNA Analyzer (Applied Biosystems, Carlsbad, CA, USA) by Sequetech DNA sequencing services (Mountain View, CA, USA). Sequence chromatograms were manually trimmed, edited, and contigs formed in Geneious Prime v.2020.2.4. All sequences were checked against GenBank using a BLAST search to confirm a match to ʻōpeʻapeʻa. Sequences produced in this study (*n* = 262) were combined with ʻōpeʻapeʻa COI sequences from (Russell et al. (2015); *n* = 59), available on GenBank (accession numbers KR350020 through KR350078). The combined dataset was aligned and trimmed by eye using MEGA-X v10.2.0 software (Kumar et al., 2018). The total COI dataset for this study consisted of 321 individuals from Hawaiʻi (H, *n* = 166), Maui (M, *n* = 92), Oʻahu (O, *n* = 47), and Kauaʻi (K, *n* = 16), spanning collection years 2005–2019 (Table S1).

A suite of 20 nuclear microsatellite loci developed for the conspecific northern hoary bat (*L. cinereus)* were amplified in ʻōpeʻapeʻa samples (Table S2) following multiplex methods for PCR and genotyping described in Korstian et al. (2013); Korstian, Hale & Williams (2015); Korstian, Hale & Williams (2014) and Pylant et al. (2016). Loci were amplified in five multiplex groups (E, F, MP1, MP2, and MP5) using a Qiagen Multiplex Reaction Kit (Qiagen, Valencia, CA, USA). Amplified microsatellite multiplexes were diluted in sample loading solution with size standard (CEQ 8000 DNA standard kit 500 Beckman Coulter or ABI DS33/LIZ500 Ladder ABI Systems) and electrophoresed on a Beckman Coulter CEQ 8000 (at UHH CGF) or an Applied Biosystems 3,730 × l Genetic Analyzer (GENEWIZ, South Plainfield, NJ, USA). Genotypes were sized and scored using Beckmann CEQ 8000 fragment analyzer package or Geneious Prime v.2020.2.4. Because fragment size assessment was conducted on two different platforms, the allele calls were standardized and checked for allelic dropout by running 8% (*n* = 24) of samples on both platforms and all microsatellite data were analyzed using Tandem (Matschiner & Salzburger, 2009), a program designed for aligning fragment sizes and merging cross platform allele bins. Genotyping errors from allelic dropout, stuttering error, and null alleles in microsatellite loci were screened using Micro-Checker 2.2.3 (Van Oosterhout et al., 2004).

Genotyping was attempted at 20 microsatellite loci for a total of 298 samples collected by island as follows: H, *n* = 131; M, *n* = 102; O, *n* = 49; and K, *n* = 16 (Tables S1, S3). We discarded the CotoG12 locus early on, as it showed presence of null alleles. Exact tests for deviations between observed (H_o_) and expected (H_e_) heterozygosity, based on Hardy-Weinberg Equilibrium (HWE), were performed in Arlequin v3.5.2.2 (Excoffier & Lischer, 2010), whereas likelihood ratio tests for genotypic linkage disequilibrium (LD) were conducted in GenePop v4.7 (Raymond & Rousset, 1995; Rousset, 2008). For both HWE and LD, Bonferonni corrections for multiple comparisons were applied to *p*-values with the p.adjust function in the R “stats” package v4.0.5 (R Core Development Team, 2020). The program FreeNA, with the ENA method (Chapuis & Estoup, 2007) corrected for positive bias in estimates of microsatellite pairwise *F*_ST_ values due to the presence of null alleles.

Mitochondrial sequences, microsatellite genotypes, and detailed tissue collection information produced through this study are available from https://doi.org/10.5066/P9COQ3ZK (Pinzari et al., 2022). The mtDNA COI sequences were also placed in the NCBI GenBank repository under accession numbers  OL894241 – OL894502.

### Genetic diversity

Patterns of genetic diversity across the four islands were inferred from ʻōpeʻapeʻa COI sequences. Per-island, average pairwise genetic distances (Dxy) and percent sequence divergence were calculated using MEGA-X v10.2.0 (Kumar et al., 2018), whereas diversity statistics number of polymorphic sites (S), haplotype diversity (Hd), per site nucleotide diversity (*π*), and Watterson’s theta (Θ) (per site and per sequence) were calculated using DnaSP v6.12.03 (Rozas et al., 2017). Those diversity statistics were calculated for individuals island and partitioned across collection years with a minimum of nine samples per time category. To account for uneven sample sizes across islands, the COI sequences were also analyzed by the minimum number of sequences available for each island (with sequence sub-sets chosen randomly). Accordingly, those datasets consisted of sequences numbering 16 (K, O, M, H), 47 (O, M, H), and 92 (M, H). Patterns of haplotype diversity were also assessed by examining relationships among collapsed unique COI haplotypes using a maximum parsimony TCS network (Clement, Posada & Crandall, 2000) and visualized using PopArt Population Analysis with Reticulate Trees (Leigh & Bryant, 2015).

Genetic diversity was assessed for nuclear microsatellites, with the number of polymorphic loci, number of alleles, expected and observed heterozygosity, gene diversity, and inbreeding coefficients calculated in Arlequin v3.5.2.2 (Excoffier & Lischer, 2010). To estimate allelic richness while accounting for uneven sample sizes, a rarefaction analysis was conducted using HP-Rare v1.1 (Kalinowski, 2005), with a minimum gene size of *n* = 32 gene copies (based on the smallest population sample size - Kauaʻi).

### Population structure and isolation by distance

The hypotheses that ʻōpeʻapeʻa populations are genetically structured by island, and that regional population samples may show genetic subdivision within islands, was tested using model and non-model based approaches on both mtDNA haplotypes and microsatellite allele frequencies. Population genetic differentiation at island-scale was assessed using pairwise *F*_ST_ and analysis of molecular variance (AMOVA) of mtDNA and microsatellite data, calculations were conducted in Arlequin v3.5.2.2 (Excoffier & Lischer, 2010). The microsatellite analysis was conducted using two datasets, observed alleles (uncorrected), and alleles corrected for null alleles using FreeNA with the ENA method (Chapuis & Estoup, 2007). Genetic structure was also assessed using two multivariate clustering methods, principal components analysis (PCA) and discriminant analysis of principal components (DAPC), implemented in the packages “ade4” v1.8 (Dray & Dufour, 2007) and “adegenet” v2.1.3 (Jombart, 2008) using R v4.1.3 (R Core Development Team, 2022). The PCA identifies components of variation that contribute the greatest percentage differences observed within the genotype data, whereas DAPC constructs linear combinations of alleles having the largest between-group variance and the smallest within-group variance (Jombart, 2008).

Genetic structure and potential for admixture was also modeled by calculating ancestry coefficients and individual sample cluster membership (K) with the program Structure v2.3.4 (Pritchard, Stephens & Donnelly, 2000). Because our total dataset of multilocus microsatellite genotypes contained uneven numbers of individuals per island, and unbalanced sampling underestimates latent population structure when using the program Structure (Puechmaille, 2016; Wang, 2017), we ran analyses for several different scenarios to alleviate potential biases: (1) by island using the total dataset while allowing for uneven sample sizes (298 individuals; *H* = 131, *M* = 101, *O* = 49, *K* = 16), and (2) with mostly even sample sizes (106 individuals; *H* = 30, *M* = 30, *O* = 30, *K* = 16). Structure parameters were set to assume admixture, correlated allele frequencies within populations (Falush, Stephens & Pritchard, 2003), and the prior for admixture, *α*, was set to vary between clusters and decreased from 1.0 to 0.1 as suggested in Wang (2017). We used a range of 1-10 for values of K and 20 iterations per K, with a burn in length of 1 ×10^5^ and 2 ×10^5^ Markov chain Monte Carlo simulations (MCMC). The interpretation of Structure output was aided by Structure Selector (Li & Liu, 2018), which computes the following measures: L(K) the maximal estimate of posterior probability for a given K; ΔK, an ad hoc estimate of the most probable number of K-clusters based on the rate of change in the log probability of the data between consecutive K-values (Evanno, Regnaut & Goudet, 2005); and supervised measures (MedMedK, MedMeanK, MaxMedK, and MaxMeanK) under three varying thresholds for ancestry coefficients (0.5, 0.6, and 0.7) as recommended in Puechmaille (2016). The ancestry coefficients were visualized across clusters using Clumpak (Kopelman et al., 2015).

Isolation by distance as a potential explanation for restricted gene flow or population structure between and within islands, was tested using a Monte-Carlo style Mantel test (with 999 replicates) for correlations between microsatellite-based matrices of Edward’s genetic distance and Euclidean geographic distances using package “adegenet” v2.1.3 in R (Jombart, 2008); and (2) regression of genetic distance ((*F*_ST_/(1- *F*_ST_)) and log transformed geographic distance in GenePop (Rousset, 2008). Isolation by distance tests were calculated at group level, represented by bats collected at 59 different collection locations across the Hawaiian Islands and within islands: *H* = 27, *M* = 21, *O* = 5, and *K* = 6.

### Historical and contemporary gene flow

The historical migration patterns of ʻōpeʻapeʻa among the islands of Hawaiʻi, Maui, and Oʻahu could include unidirectional or bidirectional movement across islands, or archipelago-wide panmixia. Using Migrate-n v.3.7.2 (Beerli, 2006; Beerli & Palczewski, 2010), we considered six different modeled migration scenarios: (1) uneven *versus* even migration rates between all pairs of islands; (2) archipelago-wide panmixia; (3) Maui as the source population of Oʻahu and Hawaiʻi populations (with no self-recruitment); (4) directional migration from Hawaiʻi to Maui and Oʻahu; (5) directional migration from Maui to Oʻahu and Hawaiʻi; and (6) directional migration from Oʻahu to Maui and Hawaiʻi. Migrate-n recommends limiting population modeling to two or three populations, thus we excluded Kauaʻi from this analysis. Microsatellite data were recoded using the amplicon length divided by repeat number (standardized in Tandem), and a Brownian motion model was used to estimate the stepwise mutation of microsatellites. Uniform prior distributions of theta were bound at 0 and 150, window size 15, and migration parameter (M) bound at 0 and 800, with window size set to 80 and the mutation rate option set relative to the data. Model runs consisted of 25 million generations, sampling every 500 generations, and using a burn-in of 6.25 million. Four variably heated chains were used following the methods of (Beerli & Palczewski, 2010). Run convergence was determined from posterior probability distributions. The Bayes factors and model probabilities were calculated using log marginal likelihoods from Bezier approximations with a custom python script provided in Beerli et al. (2019).

To provide measures of contemporary migration rates (0–2 generations previous) that may have occurred recently between Hawaiʻi, Maui, Oʻahu, and Kauaʻi, microsatellite data were analyzed using BayesAss v3.0.3 (Wilson & Rannala, 2003). Each island was treated as an individual population with posterior probability of migrant history estimated from individual ancestry measures. The program was run for 50 million generations with a burn-in of 10 million, sampling every 1,000 generations, and parameter settings as follows: step sizes of migration rate *m* = 0.1, allele frequencies *α* = 0.4, and inbreeding coefficients *f* = 0.7. Several pilot runs were conducted using varying parameters to ensure the acceptance rate of final parameters remained between 40 and 60% throughout the duration of the run. Run convergence was visualized in Tracer v 1.6 (Rambaut et al., 2018) to ensure adequate mixing through generations. The posterior probability of migrant history was inspected for each individual.

To complement the BayesAss analysis, recent migration was assessed with the gensback option (set at 2) in Structure v2.3.4 (Pritchard, Stephens & Donnelly, 2000). Sampling locations were used as priors to identify likely non-residents among a population sample or individuals that might have descended from a recent migrant. For this analysis, the number of clusters was set to *K* = 4, based on model (K) selection. The migration rate priors were varied (0.01, 0.05, 0.1) to assess sensitivity (*Pritchard & Wen, 2004*) and we used a burn-in of 200,000 and run length of 500,000 MCMC chains.

### Sex-biased dispersal testing

For carcasses that could not be reliably sexed from morphological features, genetic methods were employed to identify sex for tests of sex-biased gene flow. Of 113 samples that lacked morphological sex data, sex genotyping was performed by amplifying regions of introns on the Zfx and Zfy sex chromosomes using PCR and visualizing fragments by electrophoresis (Korstian et al., 2013; Pinzari & Bonaccorso, 2018).

Models to detect sex-biased dispersal have several assumptions, including nonoverlapping generations, dispersal of juveniles prior to reproduction, and that sampling occurred post-dispersal. Like many species, bats have overlapping generations, yet sex-biased dispersal tests have been used to explore dispersal in bats (Petit, Balloux & Goudet, 2001; Kerth, Mayer & Petit, 2002; Salgueiro et al., 2008). Because detecting sex-biased dispersal depends on the proportion of population sampled and thus greater sample size is preferred, we performed tests using microsatellite loci combined from all islands (*n* = 295, 4 groups) and for regional population samples within each island; *H* = 130, 4 groups; *M* = 99, 4 groups; *O* = 49, 2 groups; and K16, 2 groups. To compare dispersal probabilities between sexes of adults in our data, we evaluated the mean and variance assignment indices (mAI_c_, vAI_c_), *F*_ST_ and *F*_IS_, relatedness, observed heterozygosity (H_o_) and gene diversity (H_s_) measures as described in Goudet, Perrin & Waser (2002). We conducted sex bias testing with the *p* one-sided hypothesis testing option using FSTAT v2.9.4 (Goudet, 2003).

### Historical and contemporary effective population sizes

The long-term historical female (N_Ef_) and long-term (N_e_) effective population sizes were estimated using complementary mitochondrial and microsatellite DNA techniques. The mtDNA COI sequences represent the long-term historical female effective population size from tens to thousands of generations before present. These estimates were calculated with the equation *θ*=2N_e_u, with the Watterson’s estimator (*θ*) value determined using Arlequin v3.5.2.2 (Excoffier & Lischer, 2010). The per sequence per generation mutation rate, u, *(Tajima, 1993; Schenekar & Weiss, 2011)* for COI has not been published for the genus *Lasiurus*, therefore, following Korstian, Hale & Williams (2015), two different substitution rates based on cytochrome *b* in the family Vespertilionidae were applied as proxies (Nabholz, Glémin & Galtier, 2008). Adjusted to our COI sequence length (657 bp), u was set to 8.061 ×10^−6^ and 1.089 ×10^−4^. Long-term N_e_ (*i.e*., global population) size was estimated from the average expected heterozygosity (H_e_) of the microsatellite loci using two mutation models, the infinite allele model (IAM) and the stepwise mutation model (SMM). Here, N_e_ was calculated using the respective equations: SMM N_e_ = { (1/1-H)^2^-1)/8µ, and IAM N_e_ = H/(4µ(1-H) } (Nei, 1987), with mutation rates for microsatellite loci set to 10^−3^ or 10^−5^, consistent with Korstian, Hale & Williams (2015). The N_e_ values were calculated by averaging the IAM and SMM model results for each mutation rate.

The null hypothesis that per-island long-term N_Ef_ and N_e_ estimates do not differ from contemporary (recent generations) effective population size (N_eC_) was tested using the microsatellite linkage disequilibrium method in NeEstimator V2 (Do et al., 2014). The linkage disequilibrium single-sample method relates a decrease of N_e_ with an increase in genetic drift. Estimates were generated for each island with all samples over all collection years, and individual island datasets were also subdivided into temporal groupings in cases where collection year spanned a minimum two-year period sample size. Random mating was assumed and alleles with frequencies below 0.05 were removed. Estimates of N_e_ are reported with ± 95% confidence intervals determined with both jackknife and parametric re-sampling methods.

### Demographic patterns—bottlenecks and expansions

To assess whether island population demographic signatures indicate past expansions and contractions, microsatellite data were examined using a modified Garza-Williamson M-ratio test and COI sequences were subjected to tests of neutrality. The M-ratio method compares the per-locus ratio of the number of size classes with at least one observed allele to all possible allele categories calculated from the full set of allelic size ranges, with the assumption that a bottleneck event will result in loss of alleles from allele size categories. M-ratio values below a critical value of 0.68 generally indicate a recent population bottleneck (Garza & Williamson, 2001). For each island, the M-ratios were empirically calculated in Arlequin v3.5.2.2 (Excoffier & Lischer, 2010) and compared to critical values (M_c_) simulated for each island using the software Critical M (Garza & Williamson , 2001). This analysis was run by setting the probability of changes greater than one step (*ρ*g) to 0.2, the size of one-step changes (Δg) to 3.5, and varying *θ* from 0.01 to 10, which encompassed all estimates of *θ* produced by mitochondrial sequences (Busch, Waser & Dewoody, 2007.

The neutrality tests on the COI dataset were conducted using DnaSP v6.12.03 (Rozas et al., 2017). Two summary statistics, Tajima’s D (Tajima, 1989) and Fu’s F (Fu, 1997), were applied to test the null hypothesis that the number of rare and common mutations are equal, as is expected for a stable population, while the alternative is an increase in low frequency mutations consequent to a population undergoing expansion or rapid growth.

## Results

### Genetic diversity

Inter-island differences in molecular population genetic diversity were prominent in the analysis of the mtDNA COI gene, (Tables 1 and 2). The haplotype and nucleotide diversity values were variable between islands, and between years (Table 1, Table S4). Among sampled islands, Hawaiʻi showed the greatest number of unique haplotypes and polymorphic sites, while Kauaʻi showed the lowest number of unique haplotypes and fewest polymorphic sites (Table 1). Haplotype diversity ranged from 0.669 (M) to 0.125 (K) while nucleotide diversity ranged from 0.012 (M) to 0.0005 (K). The low mitochondrial diversity observed on Kauaʻi does not appear to be an artifact of sample size because results for Hawaiʻi and Maui were mostly robust across the number of rarefied sequence sets (Fig. S1A); however, nucleotide diversity observed for Oʻahu dropped considerably when sample sizes were increased (Table 1, Fig. S1B).

**Table 1:** Mitchondrial COI diversity of ʻōpeʻapeʻa by island for four Hawaiian Islands. Shown are the number of sequences analyzed in that set, number of unique haplotypes (h), number of polymorphic sites (S), haplotype diversity (Hd) with standard deviation, average nucleotide diversity (*π*) with standard deviation (SD), Wattersons theta (Θ) per site, and per sequence.

Number of sequences	Island	h	S	Hd ± SD	*π* ± SD	*θ* (site)	*θ* (seq)
16	Hawaiʻi	4	20	0.350 ± 0.148	0.003 ± 0.002	0.009	6.027
	Maui	3	23	0.633 ± 0.074	0.010 ± 0.003	0.100	6.330
	Oʻahu	3	18	0.425 ± 0.133	0.010 ± 0.003	0.008	5.420
	Kauaʻi	2	3	0.125 ± 0.106	0.0005 ± 0.0004	0.001	0.904
47	Hawaiʻi	8	6	0.378 ± 0.089	0.0007 ± 0.0002	0.002	1.358
	Maui	5	24	0.669 ± 0.037	0.012 ± 0.002	0.008	5.433
	Oʻahu	4	4	0.384 ± 0.076	0.002 ± 0.0004	0.001	0.905
92	Hawaiʻi	13	27	0.404 ± 0.065	0.001 ± 0.0006	0.008	5.700
	Maui	7	25	0.670 ± 0.023	0.012 ± 0.0013	0.007	4.908
166	Hawaiʻi	15	29	0.397 ± 0.047	0.001 ± 0.0003	0.008	5.452

**Table 2:** Genetic distances for mitochondrial COI DNA sequences in ʻōpeʻapeʻa. Average pairwise genetic distances (Dxy) and percent sequence divergence (in parentheses) for cytochrome c oxidase I (COI) in ʻōpeʻapeʻa, between and within (italicized) islands.

	Hawaiʻi	Maui	Oʻahu	Kauaʻi
Hawaiʻi	*0.473* (*0.10%*)	–	–	–
Maui	4.271 (2.47%)	*2.243* (*1.21%*)		–
Oʻahu	1.088 (0.88%)	3.534 (2.19%)	*1.367* (*1.04%*)	–
Kauaʻi	0.473 (0.10%)	4.128 (2.63%)	0.914 (0.69%)	*0.125* (*0.06%*)

The average pairwise genetic distance values ranged from 4.271 to 0.125, with differences generally greater between than within island populations, and Maui and Hawaiʻi showing the greatest spread (Table 2). The percent sequence divergence ranged from 2.63% to 0.06%, with Kauaʻi- Maui and Hawaiʻi-Maui, showing greatest differences (>2.4%).

**Figure 2:**
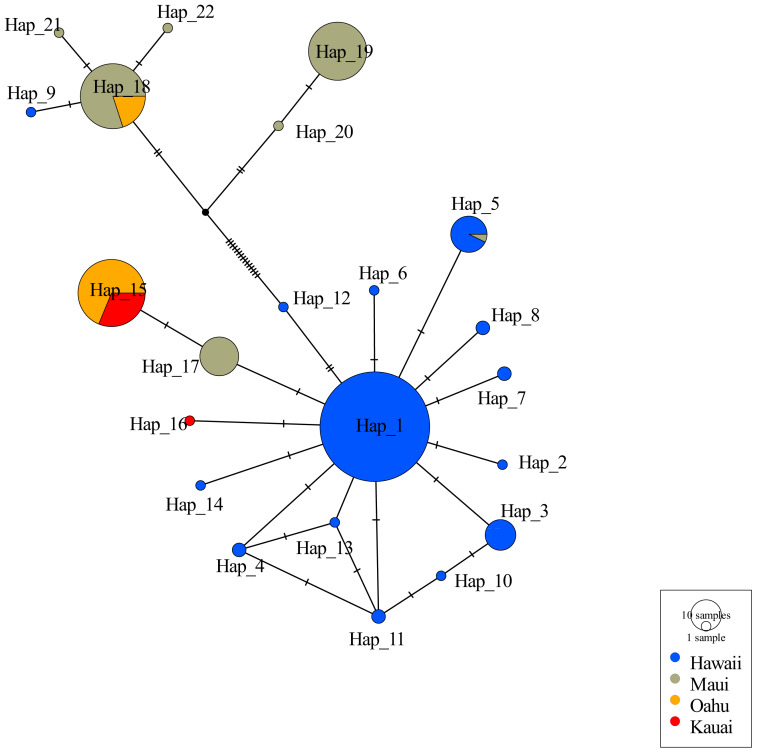
Haplotype network for 316 ʻōpeʻapeʻa across four Hawaiian Islands. Haplotype parsimony network constructed from 22 unique mitochondrial haplotypes of the CO1 region of 316 ʻōpeʻapeʻa. Unique haplotypes are represented by a colored circle, while islands are defined by separate colors. Lines with dashes between circles represent number of base pair changes between two haplotypes. Number of samples in each haplotype correspond to the size of circles in legend.

Consistent with other studies the mtDNA haplotype network indicated the presence of two clades (Fig. 2). Among the 22 unique haplotypes detected in 316 individuals, the network indicated three haplotypes were shared across islands while most were restricted to individual islands. Sequences of 5 individuals from Oʻahu (O8, O9, O14, O17, and O27) displayed heteroplasmy. None of these sequences showed evidence of contamination and all sequences were translatable into amino acids with no stop codons that would indicate they were nuclear copies of mitochondrial DNA (Numts). Most mutations were transitions and the heterozygous sites occurred across several individuals from the same collection site. Removal of these Oʻahu individuals resulted in a haplotype network with a much greater number of base pair changes, emphasizing distinctiveness of two mitochondrial clades (Fig. 2). However, when Oʻahu individuals were included, network results display a closer relationship between the two mtDNA clades (Fig. S3).

The majority of samples (*n* = 278, 93.2%) were genotyped at all 19 microsatellite loci, with a minority of samples (*n* = 20, 6.8%) genotyped at only 9–18 loci (Table S3). The number of alleles per locus ranged from 2–24 (Table S3). No evidence for consistent or significant linkage disequilibrium was found between any two loci across the four islands following Bonferroni corrections, thus all loci were considered independent, however the locus LAS8539AC was discarded, because it returned strong evidence of deviation from HWE across islands and presence of null alleles. Across the remaining 18 microsatellite loci, the per-island number of polymorphic loci ranged from 15 (O and K) to 18 (H), the per-locus and per-island mean numbers of alleles ranged from 4.93 (K) to 9.22 (H), and the observed heterozygosity between bats on different islands ranged from 0.56 (O and H) to 0.59 (M). The inbreeding coefficients (*F*_IS_) ranged from 0.013 (H) to −0.035 (O) and were not significant (*p*-value threshold 0.05), consistent with absence of the Wahlund effect within individual island datasets (Table 3). The rarified private allelic richness varied by island; 1.29 (H), 0.83 (M), and 0.25 (O and K). Partitioning data by years showed the average microsatellite diversity measure were mostly stable between time periods (Table S5).

**Table 3:** Genetic diversity in microsatellites for ʻōpeʻapeʻa on four Hawaiian Islands. Average microsatellite diversity results in ʻōpeʻapeʻa by island across 18 microsatellite loci, number of polymorphic loci (pl), rarefied allelic richness (Ar), expected heterozygosity (H_*e*_), observed heterozygosity (H_*o*_), gene diversity, inbreeding coefficients (FIS) and associated pvalues. Means are reported with standard deviations.

Island	Years	n	pl	Ar	Alleles	H_e_	H_o_	Gene diversity	*F*_IS_	*F*_IS_*p*-value
Hawaiʻi	2009–2020	131	18	6.06	9.22 ± 5.90	0.59 ± 0.29	0.57 ± 0.28	0.58 ± 0.29	0.013	0.15
Maui	1988–2020	102	16	5.71	8.23 ± 5.27	0.62 ± 0.24	0.60 ± 0.23	0.55 ± 0.28	0.008	0.29
Oʻahu	2011–2020	49	15	4.33	5.62 ± 2.57	0.56 ± 0.24	0.57 ± 0.24	0.46 ± 0.24	−0.035	0.90
Kauaʻi	2008–2019	16	15	4.16	4.93 ± 2.12	0.59 ± 0.27	0.59 ± 0.29	0.49 ± 0.26	0.003	0.50

### Genetic structure

An AMOVA test for mitochondrial haplotype differences showed significant differentiation among and within islands, with variance among islands at 67% compared to within islands at 33%, F (3, 320) = 0.669, *p* < 0.001. In contrast, the AMOVA conducted for microsatellite loci showed a very small percent of variance due to differences among islands (7.60%) or within islands (1.76%), with 90.6% of variance due to the differences within individual bats (*F* = 0.076, *p* < 0.001). Consistent with population structure, global F-statistics across all microsatellite loci were significant (p <0.001: *F*_IS_ = 0.019 [95% confidence interval (CI) [−0.005–0.04], *F*_ST_ = 0.076 [95% CI [0.05–0.09]], and *F*_IT_ = 0.093 95% CI [0.06–0.12]).

Pairwise *F*_ST_ of nuclear microsatellites were lower than those calculated using mtDNA COI, a pattern that is typical of the two marker types. However, both datasets revealed significant genetic differentiation between all island pairs (*p* < 0.05), with the exception of Hawaiʻi and Kauaʻi (Table 4) at the COI marker. The highest significant pairwise differences for COI were between Maui and Hawaiʻi and between Maui and Kauaʻi. Although *F*_ST_ values slightly decreased when corrected for null alleles using FreeNA with the ENA method, they still showed the same pattern as the uncorrected microsatellite data (Table 4, Table S6). The lowest *F*_ST_ values for the microsatellite data occurred in pairwise comparisons between Maui and Hawaiʻi, and Oʻahu and Kauaʻi.

**Table 4:** Pairwise F_*ST*_ comparisons using mitochondrial and microsatellite data among four Hawaiian Islands for all ʻōpeʻapeʻa. Pairwise FST comparisons using mitochondrial and microsatellite data among four islands for all ʻōpeʻapeʻa. Pairwise FST values are located below the diagonal with the *p*-value < 0.05 indicated by * next to the FST value; the geographic distance (kilometers) is presented above the diagonal.

	Island	Hawaiʻi	Maui	Oʻahu	Kauaʻi
Mitochondrial COI	Hawaiʻi	–	175 km	340 km	500 km
	Maui	0.780^*^	–	180 km	350 km
	Oʻahu	0.298^*^	0.467^*^	–	170 km
	Kauaʻi	−0.007	0.627^*^	0.123^*^	–
Microsatellite	Hawaiʻi	–	175 km	340 km	500 km
	Maui	0.048^*^	–	180 km	350 km
	Oʻahu	0.100^*^	0.101^*^	–	170 km
	Kauaʻi	0.103^*^	0.091^*^	0.058^*^	

### Population structure and isolation by distance

**Figure 3:**
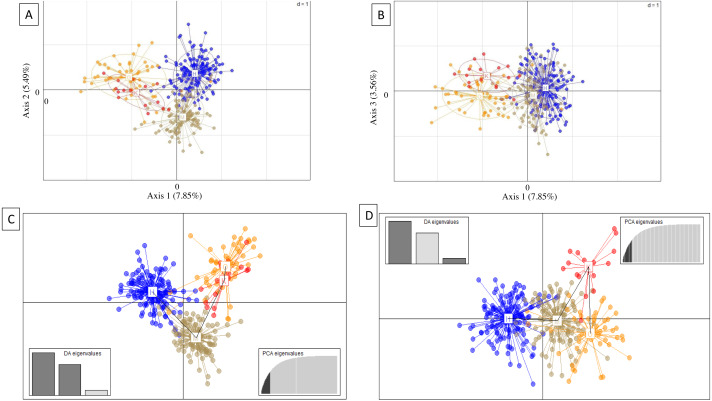
Principal components analysis (PCA) and discriminate analysis of principal components (DAPC) based on 18 microsatellite loci in ʻōpeʻapeʻa across four Hawaiian Islands. (A and B) Principal components analysis (PCA) and (C and D) discriminate analysis of principal components (DAPC) with a minimum spanning tree line illustrating distance between clusters, based on 18 microsatellite loci in ʻōpeʻapeʻa across four Hawaiian Islands, labeled as Hawaiʻi (H), Maui (M), Oʻahu (O), and Kauaʻi (K).

Genetic subdivision by island was evident from PCA and DAPC scatterplots, which showed mostly non-overlapping scatter for each island, except between Oʻahu and Kauaʻi (Fig. 3). For PCA, axis 1 (7.85% total variation) separated Oʻahu and Kauaʻi from Hawaiʻi and Maui, axis 2 (5.49% total variation) separated Maui from the other islands (Fig. 3A), and axis 1 with axis 3 (3.56% total variation) further separated Kauaʻi and Oʻahu from Hawaiʻi and Maui (Fig. 3B). For DAPC, a plot of components 1 and 2 indicated three distinct clusters with overlap between Oʻahu and Kauaʻi populations (Fig. 3C), while the plot of components 1 and 3 indicate four genetic clusters defined by island with further separation of Kauaʻi (Fig. 3D).

**Figure 4:**
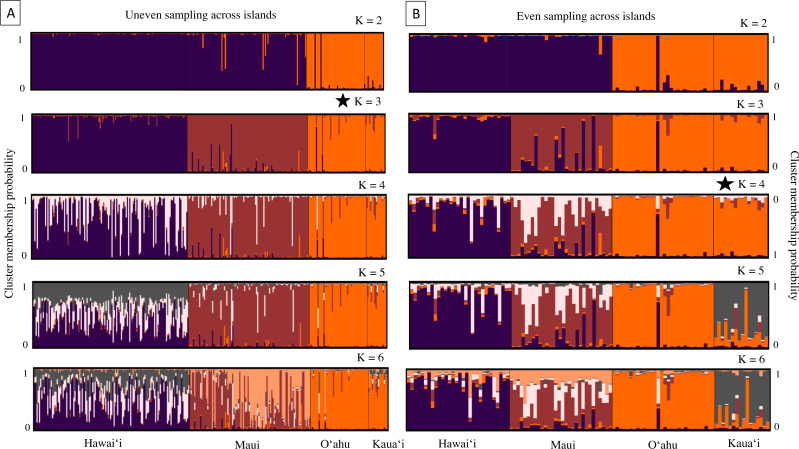
Cluster analysis results for 18 microsatellite loci in ʻōpeʻapeʻa across four Hawaiian Islands; Hawaiʻi, Maui, Oʻahu, and Kauaʻi. Bar plots from the program Structure (Pritchard, Stephens & Donnelly, 2000) showing the ancestry probabilities of individual assignment to each genetic cluster (K) for K from 1 through 6. Each vertical bar along the *x*-axis represents the genotype of an individual, grouped by their sampling island. The *y*-axis indicates the proportion of the individual’s genotype that belongs to each cluster, as represented by different colors. (A) Results from all bats sampled, where number of individuals are uneven across islands. (B) Results from a subsampled number of bats across islands, where individuals are evenly sampled across islands. The star denotes that *K* = 3 is the most likely number of genetically distinct clusters from uneven sampling, while *K* = 4 is the most likely number of clusters when sampling is more even across islands.

Results from the program Structure were concordant with PCA and DAPC scatterplots, revealing strong genetic differentiation among nearly all island populations. The number of genetic groups varied from three to four, contingent on sample datasets and based on post hoc estimators (Fig. 4). Structure results from the whole dataset supported differentiation between Hawaiʻi and Maui clusters, and a third cluster composed of individuals from both O′ahu and Kauaʻi, with Evanno and Puechmaille post hoc estimators supporting *K* = 3 (Fig. 4A). When the numbers of individuals across islands was roughly even, individuals clustered into island groups at *K* = 5 and above (Fig. 4B); however, post hoc metrics resulted in *K* = 3 for both Evanno and Puechmaille estimators. Post hoc metrics for even sampling resulted in *K* = 3 for the Evanno method but *K* = 4 for the Puechmaille estimator. When testing for potential population substructure within larger islands of Hawaiʻi and Maui, clustering patterns within Maui resulted in *K* = 2 for the Evanno method and *K* = 3 for the Puechmaille estimator. We did not find overwhelming evidence for distinct genetic clusters using these methods within the Island of Hawaiʻi. Members of the two mitochondrial clades were dispersed across the clusters identified by the Structure analysis (Fig. S4).

**Figure 5:**
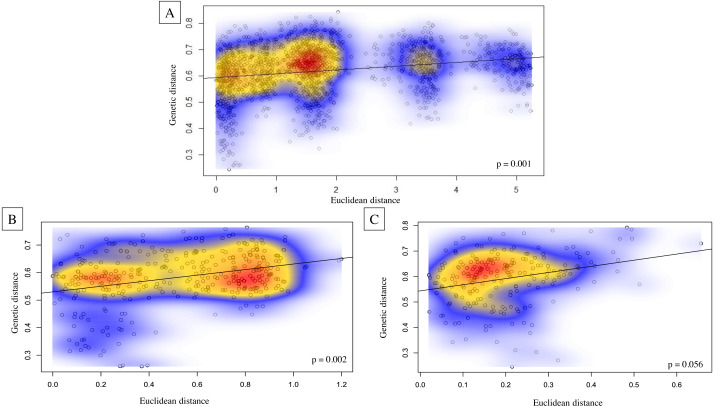
Isolation by distance Mantel tests illustrating Euclidean genetic distance between 59 geographic collection sites for microsatellite data of ʻōpeʻapeʻa across the Hawaiian Islands. Isolation by distance Mantel tests illustrating Euclidean genetic distance between 59 geographic collection sites for microsatellite data of ʻōpeʻapeʻa across the Hawaiian Islands with Kernel density estimates overlayed on correlation plots and *p*-values given in bottom right-hand corner of each graphic; (A) all four Hawaiian Islands sampled, (B) Hawaiʻi Island between 27 collection sites, and (C) Maui between 21 collection sites.

Genetic structure across all island bat populations was further corroborated by the Mantel test correlation between matrices of Edward’s genetic distance and Euclidean geographic distances across collection sites on four islands (*r* = 0.224, *p* = 0.001; Fig. 5A). Results of isolation by distance (IBD) Mantel tests conducted with adegenet (Jombart, 2008) within islands supported a relationship between genetic and geographic distance across collection sites within Hawaiʻi (*r* = 0.322, *p* = 0.002; Fig. 5B), collection sites within Maui (*r* = 0.265, *p* = 0.056; Fig. 5C), but not across collection sites within Kauaʻi (*r* = 0.260, *p* = 0.075) or O’ahu (*r* = −0.156, *p* = 0.597). Regression of genetic distance [(*F*_ST_/(1- *F*_ST_)] and log transformed geographic distance supported the alternative hypothesis that distance had an effect on genetic differentiation among collection sites across four islands (*b* = 0.04, 95% CI [0.02–0.04]), and within Maui (*b* = 0.05, 95% CI [0.03–0.10]). However, the null hypothesis could not be rejected for collection sites within Hawaiʻi, Oʻahu, or Kauaʻi.

### Dispersal and gene flow

Evidence for sex biased dispersal was inconsistent across test metrics and groups (Table 5). Across all islands, assignment indices (mAIc and vAIc), and measures of inbreeding coefficients (*F*_IS_) measures had a pattern of weak male dispersal; however, this was not significant after *p* one-sided hypothesis testing. Only samples from Maui showed significance for male biased dispersal in mAIc, vAIc, and H_S_. Within Hawaiʻi, mAIc, *F*_ST_, and *F*_IS_ measures had a pattern of female-biased dispersal, but vAIc supported male dispersal. Within Oʻahu, mAIc supported female dispersal, while vAIc and *F*_ST_ indicated male dispersal; however, *F*_IS_ supported no dispersal of either sex. Within Kauaʻi, mAIc, *F*_ST,_ and *F*_IS_ supported female dispersal. The *p* one-sided hypothesis testing was not significant for any measures within Hawaiʻi, Oʻahu, or Kauaʻi. Within Maui, male biased dispersal was supported with mAIc, vAIc, and *F*_IS_ measures, and for mAIc, vAIc, and H_S_ was significant after hypothesis testing.

**Table 5:** Sex-biased dispersal tests in ʻōpeʻapeʻa across four Hawaiian Islands and within each island. Results of sex-biased dispersal tests in ʻōpeʻapeʻa across all islands and within each island; mean assignment index (mAIC) and variance of the assignment index (vAI_*C*_), FST, FIS, relatedness (Relat), (HO), gene diversity (HS), and significance levels (*) for the *p* one-sided test.

Group		N	mAI_C_	vAI_C_	*F*_ST_	*F*_IS_	Relat	H_O_	H_S_
All islands	Females	119	0.03	20.4	0.07	0.005	0.13	0.57	0.57
	Males	176	−0.02	25.9	0.08	0.030	0.14	0.55	0.57
	*p one-sided test*		*0.477*	*0.202*	*0.913*	*0.097*	*0.881*	*0.943*	*0.856*
Hawai′i	Females	50	−0.13	23.2	−0.0017	0.026	−0.003	0.58	0.59
	Males	80	0.08	24.0	−0.0025	0.020	−0.005	0.58	0.59
	*p one-sided test*		*0.622*	*0.539*	*0.553*	*0.669*	*0.556*	*0.332*	*0.484*
Maui	Females	43	1.00	8.14	−0.0009	0.015	−0.002	0.57	0.57
	Males	56	−0.768	16.15	0.005	0.039	0.010	0.57	0.59
	*p one-sided test*		0.004*	0.037*	*0.797*	*0.184*	*0.790*	*0.362*	0.027*
O′ahu	Females	21	−0.04	21.7	0.06	−0.051	0.11	0.53	0.49
	Males	28	0.03	29.4	−0.021	−0.001	−0.043	0.49	0.49
	*p one-sided test*		*0.503*	*0.490*	*0.071*	*0.207*	*0.070*	*0.786*	*0.622*
Kauaʻi	Females	8	−0.32	5.2	−0.006	0.006	−0.01	0.50	0.50
	Males	8	0.32	5.1	0.016	−0.004	0.03	0.49	0.49
	*p one-sided test*		*0.696*	*0.635*	*0.812*	*0.676*	*0.817*	*0.508*	*0.781*

**Figure 6:**
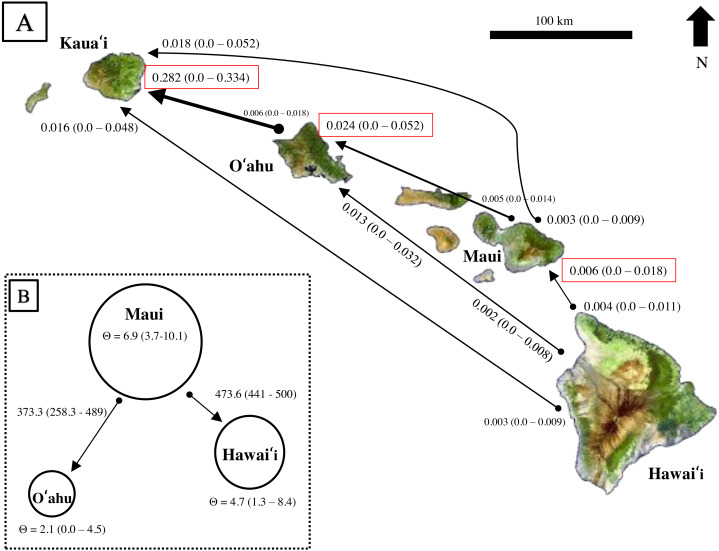
Estimates of contemporary and historical genetic migration in ʻōpeʻapeʻa between some of the Hawaiian Islands. (A) BayesAss (Wilson & Rannala, 2003) estimates of contemporary migration by ʻōpeʻapeʻa between four islands. The mean proportion of migrants based on microsatellite data is indicated next to the island with 95% confidence intervals in parentheses, while black lines connect the pairs of islands for which migration was modeled. The arrow at the tip of the line indicates a higher proportion of migrant gene flow in that direction. Red boxes highlight estimates where migrant histories were highest for that island. (B) Migrate-n (Beerli, 2006; Beerli & Palczewski, 2010) estimates of historical migration conceptualized for ʻōpeʻapeʻa between three islands and the “source–sink” model. Theta (Θ ) values calculated from Migrate-n (Θ = 4Ne µ) are represented by circle size, with Maui being the large “source” population, and Hawaiʻi and Oʻahu populations reciving migrants from Maui. Estimates of mutation-scaled historical immigration rates, from one population to the other, are shown along the arrows.

Results from BayesAss produced contemporary migration proportions, with confidence intervals including zero, thus the proportion of migrants was not significantly different from zero (Fig. 6A). Rare dispersal events may have occurred more than two generations ago, as BayesAss identified a handful of individuals on Hawaiʻi, Maui, and Oʻahu with migrant ancestry posterior probabilities, but in most cases less than 0.30 probability of being a second-generation migrant. Recent migrant ancestry results from Structure using several migration prior settings, also resulted in little to no evidence for contemporary migration (Fig. S4). Only two individuals from Oʻahu showed probability of assignment to a different island; however, the assignment probabilities were mixed across generations and populations from Hawaiʻi and Maui and decreased as migration priors increased.

Comparison of Bayes factors and model probabilities across six dispersal models from Migrate-n resulted in the probability of 1.00 for a model placing Maui as the “source” population of Oʻahu and Hawaiʻi migrants . In this scenario, Maui’s historical theta {Θ (4N_e_µ)} was ∼3 times larger than O′ahu and ∼1.4 times larger than Hawaiʻi (Fig. 6B). The mutation-scaled immigration rates (M = m/µ, m = proportion of immigrants) from Maui to Oʻahu was less than the rate from Maui to Hawaiʻi. With the mutation rate for mammalian microsatellites (µ= 1.2 ×10^−3^) from Weber & Wong (1993) applied, the rough estimate of historical migration rate from Maui to Oʻahu was 0.45 and to Hawaiʻi was 0.57 individuals per generation.

### Estimates of effective population size

Long-term historical female effective population size (N_Ef_) estimates from COI mitchondrial sequence data were similar across Hawaiʻi, Maui, and Oʻahu (min 20,796 and max 286,658, ranges contingent on mutation rate) with the Kauaʻi sample showing a smaller range (1,384–18,194; Table 6). Using methods based on heterozygosity, the long-term N_e_ estimates from microsatellite data were similar across the four islands with wide ranges and degree of overlap. However, the SMM estimates for Oʻahu and Kauaʻi were half that of Hawaiʻi and Maui (Table 6). Long term Ne was generally lower than N_Ef_ based on COI (except for the low N_e_ based on COI for Kauaʻi). The long-term N_e_ trended higher for the SMM model compared to the IAM, with N_e_ bracketing a wide range of 248 to 62,162. Estimates from averaged results between the model types for each mutation rate had a pattern of larger N_e_ for Hawaiʻi and Maui (∼490 to 49,000), and smaller N_e_ for Oʻahu and Kauaʻi (∼310 to 31,000). The average estimates using the lower mutation rate produced values that fell within the range of historical N_Ef_ estimates from mitochondrial sequences.

Linkage disequilibrium estimates of contemporary effective population sizes (N_eC_) of ʻōpeʻapeʻa across all islands and collection years were far smaller than historical long-term N_Ef_ and N_e_, but confidence intervals included infinity for Hawaiʻi and Kauaʻi, which precludes making strong inferences and indicates potentially larger N_eC_ populations (Table 7, Table S7). Estimates of N_eC_ were largest for Hawaiʻi, similar between Maui and Kauaʻi, and smallest for Oʻahu (Table 7). The Maui and Oʻahu N_eC_ estimates resulted in bounded confidence intervals. Hawai′i Island N_eC_ for annual groups in 2009–2012 and 2018–2019 were 391 and 350 respectively, while the 2018–2019 parametric confidence interval was the only metric that produced an upper bound (4,262 in Table S7). Maui’s N_eC_ for annual groups 2012–2014 and 2016–2019 were 65 and 95, respectively, and each had confidence intervals with upper bounds <500. Oʻahu’s N_eC_ for annual groups 2013–2015 and 2017–2020 were 21 and 11, respectively, with confidence interval upper bounds <50. In some cases, the sample size exceeded the N_eC_ estimate.

**Table 6:** Estimates of long-term historical female effective population size (NEf) for ʻōpeʻapeʻa on four Hawaiian islands. (A) Estimates of long-term historical female effective population size (NEf) for each island, calculated from ʻōpeʻapeʻa mitochondrial (COI) sequences based on high and low sequence mutation rates (*μ*) inferred from cytochrome b in the family Vespertilionidae. (B) Estimates of long-term global effective population size (N_*e*_) of ʻōpeʻapeʻa on four Hawaiian Islands calculated from microsatellite loci using the expected heterozygosity for two different microsatellite mutation rates (µ) (10^−3^, and 10^−5^) in both the stepwise mutation (SMM) and infinite allele (IAM) models, and an average taken between both models for each rate.

	Mutation rate	Hawaiʻi	Maui	Oʻahu	Kauaʻi
(A) Historical Female N_Ef_	High u–Low u	21,808–286,658	21,641–284,453	20,796–273,356	1,384–18,194
(B) Long Term N_e_	SMM High µ–Low µ	622–62,162	618–61,828	376–37,604	371–37,122
	IAM High µ–Low µ	361–36,099	360–35,962	251–25,052	248–24,811
	Average High and Low µ (± SD)	491 (±184)–49,131 (±18,429)	489 (±183)–48,895 (± 18,290)	313 (±89)–31,328 (±8,876)	310 (±87)–30,966 (±8,705)

**Table 7:** Estimates of contemporary effective population size (N_*e*_*C*) for ʻōpeʻapeʻa on four Hawaiian Islands. Estimates of contemporary effective population size (N_*eC*_) based on microsatellite linkage disequilibrium and 95% confidence intervals (CI) from jackknifing for ʻōpeʻapeʻa. Values were calculated with Ne Estimator V2 (Do et al., 2014). Island samples sizes are indicated with *n*.

Island	Years	*n*	N_eC_	Jacknife 95% CI	Parametric 95% CI
Hawaiʻi	2009–2020	131	1,265	314–∞	444–∞
Maui	2009–2020	98	106	55–344	80–150
Oʻahu	2011–2020	49	21	12–39	16–26
Kauaʻi	2008–2019	16	91	17–∞	29–∞

### Demographic patterns–bottlenecks and expansions

The modified Garza-Williamson M-ratios for each island population resulted in M-ratio mean values lower than the simulated range of critical M (M_c_), indicating either past population size reductions or possible bottleneck events across Maui (0.43 ± 0.20, M_c_ range [0.69–0.80]); Oʻahu (0.32 ± 0.20), M_c_ range [0.65–0.80]); and Kauaʻi (0.25 ± 0.15), M_c_ range [0.58–0.80]). For Hawaiʻi the M-ratio standard deviation encompassed critical M range (0.56 ± 0.29, M_c_ range [0.70–0.81]) The Tajima’s D neutrality test indicated a past expansion for the Hawaiʻi population, showing significant negative values across most rarefied groups (Table 8), however Maui and Oʻahu populations showed no evidence of expansions. Tajima’s D tests conducted on subsets of samples by collection year for each island, continued to support past expansion signals for Hawaiʻi for both collection periods(Table S9). The Fu’s Fs testing did not significantly support past population expansions (Tables S8, S9).

**Table 8:** Demographic characteristics for populations of ʻōpeʻapeʻa on four Hawaiian Islands. Tajimas D demographic characteristics by island for ʻōpeʻapeʻa subsampled to rarify the number of cytochrome c oxidase (COI) mitochondrial sequences.

Number of sequences	Hawaiʻi	Maui	Oʻahu	Kauaʻi
16	−2.35^*^	−0.09	1.19	−1.69
47	−1.71	1.62	1.17	–
92	−2.60^*^	1.87	–	–
166	−2.54^*^	–	–	–

**Notes.**

* *P* < 0.001.

## Discussion

For an endangered species of unknown census size and dispersal capabilities, knowledge of population genetic diversity, structure, gene flow, and effective population size can help inform management decisions aimed at mitigating the loss of individuals due to threats. Our analyses of mitochondrial sequences and microsatellite loci for ʻōpeʻapeʻa supported an overarching pattern of structure by island with differentiated populations on at least three Hawaiian Islands, a weak signature of genetic isolation by geographic distance and little to no contemporary gene flow between islands. Measures of sex-biased dispersal and the estimated size of contemporary effective genetic populations each varied by island. Our findings do not support the hypothesis that two mtDNA lineages are reproductively isolated, rather that patterns of genetic differentiation at nuclear markers are dominated by island of origin. This lack of correspondence between microsatellite ancestry coefficients and mitochondrial haplotype is consistent with a single species across the Hawaiian archipelago.

### Genetic diversity

Insular patterns from mitochondrial and nuclear loci generally mirrored heterozygosity values reported for single nucleotide polymorphisms in (Pinzari et al., 2020), with higher diversity values reported for Maui, and lower diversity values reported for Hawaiʻi and Oʻahu. Our mitchondrial haplotype network results, which included only individuals from the Hawaiian Islands, were similar to the mitochondrial clade networks described in phylogenetic studies of Russell et al. (2015) and Baird et al. (2017) when individuals with point mutations suggestive of heteroplasmy were removed. Our increased sampling and geographic coverage presented 11 additional haplotypes. One clade occurred across four islands, although it was most abundant on Hawaiʻi. The second clade, most abundant on Maui, also was present in low numbers on Oʻahu and Hawaiʻi. Peak mitochondrial haplotype diversity on Maui reported by Russell et al. (2015) and Baird et al. (2017) was supported by our results. Sampling to include Lanai and Molokaʻi Islands may uncover additional diversity increasing our understanding of the unique haplotype dynamics occurring on Maui.

The presence of heteroplasmy in five individuals from Oʻahu is unique among Hawaiian bats, although heteroplasmy occurs at high rates in vespertilionid bats elsewhere (Petri, Von Haeseler & Pääbo, 1996; Wilkinson et al., 1997). Heteroplasmy may result from paternal mtDNA leakage, or as a function of de novo somatic mutations, or from maternal transmission *via* heteroplasmic eggs. Pinzari et al. (2020) reported the highest number of non-synonymous mutations within Oʻahu bats, thus observations in our study could result from mutational processes or oxidative mutations linked to infection or stressful reproductive effort (note that four of five individuals in our study were males) (Jebb et al., 2018).

In comparison to northern hoary bat populations, ʻōpeʻapeʻa exhibited low heterozygosity with similar levels among islands. Also, fewer alleles per loci occur in ʻōpeʻapeʻa compared to the northern hoary bat (Korstian, Hale & Williams, 2014; Korstian, Hale & Williams, 2015; Keller et al., 2014). A caveat is that downward bias of genetic diversity estimates from microsatellite primers transferred across species may occur when a small number of loci are selected based on high polymorphism in the focal species because congeners may have fixed or relatively low polymorphisms (Allendorf, Luikart & Aitken, 2012). Alternatively, it is possible that fewer alleles in the ʻōpeʻapeʻa population is due to founder effects and subsequent genetic drift as the ancestral population expanded. If populations are approaching or have reached migration-drift equilibrium, genetic differentiation within islands is not expected to be greater than differentiation among islands. Drift is strongest in small populations and thus may explain the lower genetic diversity on small islands like Oʻahu and Kauaʻi. Isolated populations also may show reduced allelic richness and gene diversity (Broquet et al., 2010), which aligns with those measures for Oʻahu and Kauaʻi and the geographic distance of those islands from the others.

### Population structure

Evidence of population structure using both mtDNA and microsatellite markers is absent in localized studies of *L. cinereus* (Korstian, Hale & Williams, 2015; Pylant et al., 2016), thus indicating substantial exchange of genetic material *via* dispersal and mating opportunities (Waples & Gaggiotti, 2006). In contrast, population genetic structure might be expected in island bat species where dispersal is limited by broad oceanic channels and/or resources are annually abundant within an island. Genetic isolation of island bat populations descending from continental populations has been demonstrated for molossids and vespertilionids, having large open water crossings as barriers to gene flow; examples include *Myotis punicus* in the Mediterranean (Biollaz et al., 2010), *Nyctalus azoreum* in the Azores (Salgueiro et al., 2004; Salgueiro et al., 2007; Salgueiro et al., 2008), *Tadarida brasiliensis* in the Bahamas (Speer et al., 2017), and two species of *Miniopterus* in Madagascar (Weyeneth et al., 2011). We found a weak signature of IBD across the four islands in this study and within Hawaií and Maui, indicating that geographic distance may restrict dispersal in ʻōpeʻapeʻa to some extent. Although heavily sampled, there was not strong support for IBD within Hawaiʻi from both methods used. Our number of sampling sites for Oʻahu and Kauaʻi were likely too few to enable detection of such patterns.

Nuclear microsatellite clustering in Structure analyses adds additional evidence to an overarching pattern of island structure for ʻōpeʻapeʻa reported with SNPs in Pinzari et al. (2020). In this study, three clusters have a clear signal using data from all available individuals. However, analysis conducted using similar numbers of individuals per island revealed that a greater number of genetic clusters could be present with sub-structure within Maui evident and that Kauaʻi was distinct from other islands. Increased sampling density could better test hypotheses of regional population structure within Oʻahu and Kauaʻi. Results from Bayesian clustering programs like Structure can overestimate genetic structure when IBD is also present across a continuously distributed population (Frantz et al., 2009; Perez et al., 2018); however, this may have limited effect on our results because ʻōpeʻapeʻa populations are strongly structured by island and the signal of IBD was not strong.

Estimates of genetic differentiation for nuclear microsatellites maintained significance in population structure between islands but were subtler than mitochondrial results. The large values of population structure estimated from mtDNA may be due to the smaller effective population size of mtDNA resulting in greater effects of genetic drift on population structure for mtDNA (Allendorf, Luikart & Aitken, 2012). The latter may occur when populations have been isolated over short periods of time (Zink & Barrowclough, 2008). In addition microsatellites may underestimate *F*_ST_ if high mutation rates cause size homoplasy even if migration rates are low (Balloux et al., 2000).

### Migration history and gene flow

Model estimates of historical connectivity indicate that dispersal throughout Maui, Hawaiʻi, and Oʻahu may have always been limited; for example, if estimated movements were less than 1 individual per generation. However, these results present a challenge for comparisons due to the differing model assumptions, especially if the true scenario of island migration is not captured in the historical Migrate-n models. For example, during times of lower sea level when the regional ‘Maui Nui super-island complex’ was contiguous (Price & Elliot-Fisk, 2004), genetic separation would not have been maintained by spatial distance or large oceanic channel barriers. Our Migrate-n models selected a model that placed Maui as the source of Hawaiʻi and Oʻahu migrants. This model result and genetic diversity values, are additional evidence that Maui represents the founding population for Hawaiian ʻōpeʻapeʻa as hypothesized in Russell et al. (2015); Baird et al. (2017), and Pinzari et al. (2020). Additionally, historical patterns of gene flow in ʻōpeʻapeʻa contrast with the biogeographical stepping-stone model or “progression rule” of colonization modeled to occur across the Hawaiian archipelago. In this model, Kauaʻi is the oldest of islands that formed along the volcanic hot spot (Shaw & Gillespie, 2016; Wagner & Funk, 1995). Contemporary migration estimates from microsatellites using BayesAss did not support gene flow between islands over recent generations, nor did we detect recent migrants using alternative methods in Structure. Although the estimates of recent movement between islands we generated were not significant, even a few individuals moving between populations per generation could maintain levels of gene flow. It appears that dispersal events between islands are incredibly rare for ʻōpeʻapeʻa, but could potentially be triggered by extreme weather events, *e.g.* (such as hurricanes) (Fleming & Murray, 2009). It is also possible that the number of bats and the time intervals sampled were not broad enough to detect long term temporal gene flow patterns.

Diversity differences found between microsatellite loci and mtDNA may indicate sex-biased dispersal in bat species, in addition to inherent differences between mtDNA and nuclear DNA patterns of divergence (Moussy et al., 2013; Petit, Balloux & Goudet, 2001). Male-biased dispersal occurs in forest and crevice dwelling vespertilionid bat species including, *Myotis bechsteinii* (Kerth, Mayer & Petit, 2002), *Myotis septentrionalis* (Arnold, 2007), and *Vespertilio murinus* (Safi, König & Kerth, 2007). Female-biased dispersal has been documented in *Nyctalus azoreum* in the Azores Salgueiro et al. (2008). If female bats return to the same roost areas each breeding season and dispersal is male-biased, this will result in mtDNA showing greater population differentiation than microsatellites. We found greater population differentiation in mtDNA, potentially indicating a pattern of female philopatry to islands. However, sex-biased hypothesis testing revealed no significant sex-biased dispersal across or within Hawaiʻi, Oʻahu, and Kauaʻi for either sex. On Maui, our data did support male-biased sex dispersal. Sex-biased tests have limited performance when dispersal rates are low, and limited power in cases where the bias in dispersal is greater than 80:20 (Goudet, Perrin & Waser, 2002). When dispersal is less than 10%, the vAIc statistic can be informative, but normally *F*_ST_ is considered robust to changes in sampling and magnitude, whereas mAIc has been described as a measure between the two (Goudet, Perrin & Waser, 2002). Our vAIc statistic supported male-biased dispersal patterns within Hawaiʻi and Oʻahu but *F*_ST_ and mAIc metrics did not align and conversely indicated potential female-biased dispersal. The proportion of the population and number of individuals sampled strongly influences the power to detect sex-biased dispersal, thus our limited sampling on Oʻahu and Kauaʻi may not be sufficient to detect true patterns within those islands.

Although radio tracking studies on Hawaiʻi and Maui have demonstrated varying scales of nightly movement for both sexes of ʻōpeʻapeʻa (Bonaccorso et al., 2015; HT Harvey and Associates, 2020), no telemetry studies have demonstrated seasonal or long-range movements within or between islands. Seasonal movements have thus far have been inferred from acoustic studies across elevational gradients without a sex specific context for interpretation (Menard, 2001; Gorresen et al., 2013; Bonaccorso et al., 2016; Todd, Pinzari & Bonaccorso, 2016; West Inc, 2020; West Inc, 2021). The contrasting signals of possible female sex-biased dispersal in our results, are difficult to untangle from sex-biased dispersal because of testing limitations and panmixia effects within islands and warrant future investigation. Continued field-based capture studies focusing on female roost fidelity, the identification of seasonal roosts, swarming (mating) sites, and examination of genotypes at more distant collection sites within islands could contribute to a greater understanding of mating systems, gene flow, and sex-specific dispersal patterns in ʻōpeʻapeʻa.

### Estimates of genetic effective population size

Extinction risk can be predicted if both population census size, genetic effective population size, and levels of gene flow are known (Luikart et al., 2010). Although current census population sizes of ʻōpeʻapeʻa on each Hawaiian island remain unknown and confidence intervals are wide, estimates of contemporary genetic effective population sizes from this study indicate that the Oʻahu and Maui populations may be lower in abundance than those of Hawaiʻi and Kauaʻi. Effective population size estimates aid in evaluating conservation programs for endangered species as they may flag inbreeding depression and potential loss of adapability to environmental change and disease (Waples, 2002; Funk et al., 2019; Hohenlohe, Funk & Rajora, 2021). Methods that infer effective population size assume simple population models; however, migration, spatial structure, reproductive success, and overlapping generations can confound results (Waples & England, 2011). Effective population estimates are not equivalent to the true “census” population size, and all estimates we report here should be interpreted with caution and subject to change with additional data. Thresholds for population viability, known as the 100/1000 rule (Franklin, 1980; Frankham, Bradshaw & Brook, 2014), state that a population should have an N_e_ of at least 100 individuals to avoid inbreeding depression, and an N_e_ of at least 1,000 to avoid erosion in evolutionary potential. Using the linkage-disequilibrium (LD) method, contemporary estimates of genetic effective population size (N_eC_) vary by island, with the Hawai′i estimate exceeding 1,000 individuals. On Hawaiʻi and Kauaʻi estimates were imprecise having infinity as an upper bound in confidence, and indicating N_eC_ could be greater than 500, as the LD method is known to be more precise when effective population size is <500 (Gilbert & Whitlock, 2015). We estimated N_eC_ with bounded CI to be <500 on Maui and <40 on Oʻahu, thus management actions that reduce mortality from threats, such as collisions with wind turbines, are important to the long-term persistence of hoary bat populations on those islands (Frick et al., 2017; Friedenberg & Frick, 2021). Reductions in genetic diversity caused by population decline, disconnection, or increased inbreeding, to either the highly diverse Maui population or the differentiated Oʻahu and Kauaʻi populations could erode the total genetic diversity and adaptive potential of ʻōpeʻapeʻa across the Hawaiian Islands.

Statistical estimation techniques of contemporary effective population sizes using the LD method are prone to uncertainty, in species such as bats with overlapping generations (Waples, Antao & Luikart, 2014). However, Waples & Do (2010) suggest that estimates using LD on population samples that include multiple cohorts, as we have presented, may approximate N_e__C_, if the population sample included enough cohorts to encompass one generation. Although generation time for ʻōpeʻapeʻa is unknown, we assume generation time to be at least two years whereas our population samples spanned nearly a decade. An alternative model for species with overlapping generations is to use a single-sample method based on parentage (Wang, 2009), which requires the age and sex for each individual. New DNA methylation techniques show promise for estimating generation time and age estimation in bats (Wilkinson et al., 2021). Additionally, utilizing single nucleotide polymorphisms from a larger number of individuals may refine estimates of ʻōpeʻapeʻa effective population sizes, as was recently examined for *L. cinerus* by Cornman et al. (2021).

Long-term effective population estimates for all ʻōpeʻapeʻa sampled in this study produced a range of 248 to 62,162 bats. We reported both SMM and IAM models using high, average, and low mutation rates because evolutionary mutation rates can vary within and across loci. These averaged models produced similar estimates for each island and indicate that long term maintenance of genetically diverse populations may require several hundred to tens of thousands of ʻōpeʻapeʻa. Historical female genetic effective population estimates for ʻōpeʻapeʻa presented wider ranges of 1,384 to 286,658 bats; however, the precision of these estimates is unknown because confidence intervals cannot be calculated. Molecular population genetic estimates of genetic effective population size between historical female (N_Ef_) and long-term N_e_ overlap when confidence intervals of the latter are taken into consideration. Historical genetic effective population sizes likely reflect the ancestral high genetic diversity of founding *L. cinereus* individuals from a large continental population.

### Demographic changes

Population bottlenecks are distinguished by loss of rare alleles, which typically has little effect on heterozygosity. When the population size or genetic effective size is reduced, inbreeding leads to an excess of common microsatellite alleles compared to the number of rare alleles expected under equilibrium conditions (Cornuet & Luikart, 1996). The M-ratio test indicated genetic bottlenecks with different intensity may have occurred across Maui, Oʻahu, and Kauaʻi. These measures do not detect or reflect possible recent declines in the abundance of ʻōpeʻapeʻa because genetic diversity measures lack the power to detect changes over short time spans for species with large effective population sizes, high pre-bottleneck diversity, or high connectivity (Peery et al., 2012). Genetic bottlenecks also can retain the signature of decline over many generations and be a product of population disconnection (*e.g.*, establishing a population on a new island) rather than exclusive of reduction in population size or effective size (Broquet et al., 2010). Population growth can be signaled by significant negative Tajima’s D values, high haplotype diversity, and low sequence diversity. Tajima’s D was significantly negative for Hawaiʻi, indicating past signatures of population expansion and is congruent with our migration history, gene flow, and haplotype results. Signatures of population expansions using coalescent modeling were detected in ʻōpeʻapeʻa mitochondrial clades at 800, 10,000 (Russell et al., 2015), and 20,000 years before present (Baird et al., 2017), consistent with population expansion on Hawaiʻi and supported by the high density of minor alleles in site frequency spectra from Pinzari et al. (2020). The power to detect bottlenecks would be greatly improved with demographic coalescent methods as our current results do not offer a context on when in the past a bottleneck occurred for each the island. Incorporating samples from ʻōpeʻapeʻa in museum collections prior to 2005 could enable more accurate evaluations of bottlenecks and/or declines in genetic diversity that may have occurred in the last two centuries, as well as refine estimates of historical population sizes.

## Conclusions

‘Ōpe‘ape‘a populations are genetically structured by island and contemporary genetic effective population size estimates are consistent with larger populations on Hawaiʻi and Kauaʻi relative to Oʻahu and Maui. The historical effective population size estimates demonstrate that ʻōpeʻapeʻa have retained minor amounts of ancestral genetic diversity since the founding individuals arrived and expanded across the Hawaiian Islands. The small estimates of contemporary effective population size, coupled with little to no contemporary gene flow between islands, indicates that genetically distinct island populations may directly benefit from conservation actions aimed at the individual island level. Although solitary, foliage roosting, migratory bats cannot be easily censused, we can begin to understand key aspects of their demographic histories, movements, and population patterns using molecular population genetic tools. Management actions aimed at reducing mortality from threats, including collisions with wind turbines, may be important to maintaining adaptive genetic potential and long-term persistence of ʻōpeʻapeʻa populations in Hawaiʻi.

##  Supplemental Information

10.7717/peerj.14365/supp-1 Supplemental Information 1 Number of mitochondrial sequences (mtDNA) and microsatellite genotypes (nDNA) obtained from male and female ʻōpeʻapeʻa (Hawaiian hoary bat: *Lasiurus semotus*) collected on four islands from 1988–2020 Female (F), male (M), unknown (U). MtDNA numbers include 262 sequences from the current work plus 59 sequences obtained from Russell et al. (2015); *n* = 59), available on GenBank (Accession numbers KR350020.1 through KR350078.1).

10.7717/peerj.14365/supp-2 Supplemental Information 2 Primer details of 20 microsatellite loci used for multiplex genotype testing in ʻōpeʻapeʻa (Hawaiian hoary bat: *Lasiurus semotus*). Repeats, size range, and number of alleles represent those found with the loci in northern hoary bats (*Lasiurus.cinereus*) from *Korstian et al.* (*2015*) and *Keller et al.* (*2014*).

10.7717/peerj.14365/supp-3 Supplemental Information 3 Microsatellite characteristics for 20 loci tested on ʻōpeʻapeʻa (Hawaiian hoary bat: *Lasiurus semotus*) across four Hawaiian islands. Number of individuals amplified at locus (n), number of alleles (n_*a*_), allelic richness (Ar), observed (H o) and expected heterozygosity (H_*e*_), significance level of Hardy-Weinberg (HWE) tests, null alelle frequencies based on *Van Oosterhout et al.* (*2004*), and inbreeding coefficient (F_*IS*_). The term mono is used to describe loci that were monomorphic.

10.7717/peerj.14365/supp-4 Supplemental Information 4 Mitchondrial COI diversity and demographic characteristics for ʻōpeʻapeʻa (Hawaiian hoary bat: *Lasiurus semotus*) by island and a subset of bats pooled into a collection year period Number of sequences obtained (n), number of haplotypes (H), number of polymorphic sites (S), haplotype diversity (Hd) with standard deviation (SD), nucleotide diversity (*π*), Watterson’s theta estimator (*θ*) per site and per sequence.

10.7717/peerj.14365/supp-5 Supplemental Information 5 Average microsatellite diversity results by collection year period for ʻōpeʻapeʻa (Hawaiian hoary bat: *Lasiurus semotus*) across three Hawaiian Islands Sample size (n), number of polymorphic loci, allelic richness (Ar), mean number of alleles, mean expected heterozygosity (H_*e*_), mean observed heterozygosity (H_*o*_), average gene diversity, and population inbreeding coefficient (F_*IS*_). Means are reported with standard deviations.

10.7717/peerj.14365/supp-6 Supplemental Information 6 Pairwise F_*ST*_ values for microsatellites in ʻōpeʻapeʻa (Hawaiian hoary bat: *Lasiurus semotus*) adjusted for null alleles Pairwise F_*ST*_ values for microsatellites in ʻōpeʻapeʻa (Hawaiian hoary bat: *Lasiurus semotus*) adjusted for null alleles using the ENA method in FreeNA (*Chaupis & Estoup, 2007*) are located below the diagonal, while associated 95% confidence intervals are given in brackets above the diagonal.

10.7717/peerj.14365/supp-7 Supplemental Information 7 Additional estimates of contemporary effective population size (N_*eC*_) for ʻōpeʻapeʻa (Hawaiian hoary bat: *Lasiurus semotus*) Additional estimates of contemporary effective population size (N_*eC*_) by collection year period based on microsatellite linkage disequilibrium and 95% confidence intervals (CIs) from jackknife and parametric resampling for ʻōpeʻapeʻa (Hawaiian hoary bat: *Lasiurus semotus*). Values were computed with Ne Estimator V2 (Do et al., 2014).

10.7717/peerj.14365/supp-8 Supplemental Information 8 Fu’s Fs values for ʻōpeʻapeʻa (Hawaiian hoary bat: *Lasiurus semotus*) by island rarified to the number of cytochrome c oxidase I (COI) mitochondrial sequences available for each island Fu’s Fs values for ʻōpeʻapeʻa (Hawaiian hoary bat: *Lasiurus semotus*) by island rarified to the number of cytochrome c oxidase I (COI) mitochondrial sequences available for each island.

10.7717/peerj.14365/supp-9 Supplemental Information 9 Tajima’s D and Fu’ss Fs values for ʻōpeʻapeʻa (Hawaiian hoary bat: *Lasiurus semotus*) from cytochrome c oxidase I (COI) mitochondrial sequences by island and collection year period. ** *P* < 0.001, * *P* < 0.001 Tajima’s D and Fu’s Fs values for ʻōpeʻapeʻa (Hawaiian hoary bat: *Lasiurus semotus*) from cytochrome c oxidase I (COI) mitochondrial sequences by island and collection year period. ** *P* < 0.001, * *P* < 0.001

10.7717/peerj.14365/supp-10 Supplemental Information 10 Mitochondrial COI diversity for ʻōpeʻapeʻa (Hawaiian hoary bat: *Lasiurus semotus*) by island for four Hawaiian Islands and rarified to sampling of DNA sequences (A) number of haplotypes (h), number of polymorphic sites (S) appear above bars in brackets, mean haplotype diversity (Hd). (B) mean nucleotide diversity (*π*), Watterson’s theta per site (bold **x**), and Watterson’s theta per sequence in brackets above bars. Means are reported error bars that represent standard deviations.

10.7717/peerj.14365/supp-11 Supplemental Information 11 Geographic representation of mitochondrial COI haplotypes for ʻōpeʻapeʻa (Hawaiian hoary bat: *Lasiurus semotus*) across four Hawaiian Islands Pie charts represent the haplotype frequencies detected at each collection site. Size of the circle reflects the number of individuals from that collection site, larger circles indicate more individuals collected.

10.7717/peerj.14365/supp-12 Supplemental Information 12 Haplotype parsimony network constructed from 21 unique mitochondrial haplotypes of the CO1 region of 321 ʻōpeʻapeʻa, including 5 individuals from Oʻahu with potential heteroplasmy Haplotype parsimony network constructed from 21 unique mitochondrial haplotypes of the CO1 region of 321 ʻōpeʻapeʻa (Hawaiian hoary bat: *Lasiurus semotus*), including 5 individuals from Oʻahu with potential heteroplasmy. Unique haplotypes are represented by a colored circle, while islands are defined by separate colors. Lines with dashes between circles represent number of base pair changes between two haplotypes. Number of samples in each haplotype correspond to circle sizes in legend.

10.7717/peerj.14365/supp-13 Supplemental Information 13 Mitochondrial clades overlaid on island populations defined by STRUCTURE analysis with potential migrant ancestry Distribution of mitochondrial clade and potential identification of migrant ancestry for individual ʻōpeʻapeʻa (Hawaiian hoary bat: *Lasiurus semotus*) over ancestry membership in program Structure (Pritchard, Stephens & Donnelly, 2000) plots for Hawaiʻi, Maui, Oʻahu, and Kauaʻi.

10.7717/peerj.14365/supp-14 Supplemental Information 14 Microsatellite data for Hawaiian hoary bats Data from 298 individuals at 18 microsatellite loci

10.7717/peerj.14365/supp-15 Supplemental Information 15 Hawaiian hoary bat tissue collection details Collection details for each individual, coordinates and groupings for IBD testing, groupings for sex biased dispersal testing.

## Additional Information and Declarations

### Field Study Permissions

The following information was supplied relating to field study approvals (*i.e.*, approving body and any reference numbers): Biological samples from bats were collected under federal (US Fish and Wildlife Service Threatened and Endangered Species Permit: TE003483) and state (State of Hawaiʻi Division of Forestry and Wildlife, Division of Forestry and Wildlife Collection Permit: DLNR-DOFAW WL05-03 through WL19-52) endangered species collection permits.

### Data Availability

The following information was supplied regarding data availability: The mitochondrial DNA sequences, microsatellite allele fragment data and tissue collection details are available at US Geological Survey Science Base: Pinzari, C.A., Bellinger, M.R., Price, D.K., and Bonaccorso, F.J., 2022, Hawaiian hoary bat population genetics 1988 to 2020: U.S. Geological Survey data release, https://doi.org/10.5066/P9COQ3ZK. The mitochondrial CO1 DNA sequences and microsatellite data are available in the Supplementary Files; and the DNA sequences are available at GenBank:  OL894241 to  OL894502.
