# Supplementary material for: Genetic diversity, structure, and effective population size of an endangered, endemic hoary bat, ʻōpeʻapeʻa, across the Hawaiian Islands
Source: PeerJ. 2023 Jan 25;11:e14365. doi: 10.7717/peerj.14365 (PMC9884036; doi:10.7717/peerj.14365)
Supplement: Supplemental Information 1 — Female (F), male (M), unknown (U). MtDNA numbers include 262 sequences from the current work plus 59 sequences obtained from Russell et al. (2015); n = 59), available on GenBank (Accession numbers KR350020.1 through KR350078.1). [file peerj-11-14365-s001.docx]

|  |  |  | mtDNA | | | nDNA | | | |
| --- | --- | --- | --- | --- | --- | --- | --- | --- | --- |
| Island | Year | F | | M | Total | F | M | U | Total |
| Hawai‘i | 2005 | 1 | | 3 | 4 | - | - | - | - |
|  | 2006 | 6 | | 1 | 7 | - | - | - | - |
|  | 2007 | - | | 1 | 1 | - | - | - | - |
|  | 2008 | 1 | | 1 | 2 | - | - | - | - |
|  | 2009 | 7 | | 10 | 17 | 1 | 3 | - | 4 |
|  | 2010 | 21 | | 19 | 40 | 12 | 16 | - | 28 |
|  | 2011 | 4 | | 5 | 9 | 5 | 5 | - | 10 |
|  | 2012 | 1 | | 1 | 2 | 1 | 1 | 1 | 3 |
|  | 2013 | 3 | | 2 | 5 | 3 | 2 | - | 5 |
|  | 2014 | - | | 1 | 1 | - | 1 | - | 1 |
|  | 2015 | 2 | | 4 | 6 | 2 | 4 | - | 6 |
|  | 2016 | 1 | | 1 | 2 | 1 | 1 | - | 2 |
|  | 2017 | 2 | | - | 2 | 2 | - | - | 2 |
|  | 2018 | 4 | | 12 | 16 | 5 | 12 | - | 17 |
|  | 2019  2020 | 16  - | | 36  - | 52  - | 16  1 | 36  - | -  - | 52  1 |
|  | All Years | 69 | | 97 | 166 | 49 | 81 | 1 | 131 |
| Maui | 1988 | - | | - | - | - | - | 1 | 1 |
|  | 1989 | - | | - | - | 1 | - | - | 1 |
|  | 1999 | - | | - | - | - | 1 | 1 | 2 |
|  | 2009 | 2 | | - | 2 | 2 | - | - | 2 |
|  | 2010 | 3 | | 2 | 5 | 3 | 2 | - | 5 |
|  | 2011 | 2 | | 1 | 3 | 2 | 1 | - | 3 |
|  | 2012 | - | | 1 | 1 | - | 1 | - | 1 |
|  | 2013 | 4 | | 6 | 10 | 4 | 6 | - | 10 |
|  | 2014 | 5 | | 3 | 8 | 5 | 3 | - | 8 |
|  | 2015 | - | | 1 | 1 | - | 3 | - | 3 |
|  | 2016 | 5 | | 8 | 13 | 5 | 8 | - | 13 |
|  | 2017 | 9 | | 11 | 20 | 9 | 14 | - | 23 |
| O‘ahu | 2018 | 7 | | 11 | 18 | 7 | 11 | - | 18 |
|  | 2019 | 7 | | 4 | 11 | 6 | 4 | - | 10 |
|  | 2020 | - | | - | - | - | 2 | - | 2 |
|  | All Years | 44 | | 48 | 92 | 44 | 56 | 2 | 102 |
|  | 2011 | 2 | | - | 2 | 2 | - | - | 2 |
|  | 2012 | 2 | | 1 | 3 | 2 | 1 | - | 3 |
|  | 2013 | 3 | | 10 | 13 | 3 | 10 | - | 13 |
|  | 2014 | 4 | | 6 | 10 | 4 | 6 | - | 10 |
|  | 2015 | 1 | | 6 | 7 | 1 | 6 | - | 7 |
| Kaua‘i | 2016 | 2 | | 1 | 3 | 2 | 1 | - | 3 |
|  | 2017 | 2 | | 1 | 3 | 3 | 1 | - | 4 |
|  | 2018 | 4 | | 2 | 6 | 4 | 2 | - | 6 |
|  | 2020 | - | | - | - | - | 1 | - | 1 |
|  | All Years | 20 | | 27 | 47 | 21 | 28 | - | 49 |
|  | 2008 | - | | 1 | 1 | - | 1 | - | 1 |
|  | 2009 | - | | 1 | 1 | - | 1 | - | 1 |
|  | 2015 | 1 | | - | 1 | 1 | - | - | 1 |
|  | 2019 | 7 | | 6 | 13 | 7 | 6 | - | 13 |
|  | All Years | 8 | | 8 | 16 | 8 | 8 | - | 16 |
|  | TOTALS | 141 | | 180 | 321 | 122 | 173 | 3 | 298 |
