# Supplementary material for: Genetic diversity, structure, and effective population size of an endangered, endemic hoary bat, ʻōpeʻapeʻa, across the Hawaiian Islands
Source: PeerJ. 2023 Jan 25;11:e14365. doi: 10.7717/peerj.14365 (PMC9884036; doi:10.7717/peerj.14365)
Supplement: Supplemental Information 2 — Repeats, size range, and number of alleles represent those found with the loci in northern hoary bats (Lasiurus.cinereus) from Korstian et al. (2015) and Keller et al. (2014). [file peerj-11-14365-s002.docx]

| *Group* | *Locus Name* | *Forward Primer or Tailed Forward Primer*  *+ Flurophore: Tail* | *Reverse Primer* | *Repeat* | *Size Range (bp)* | *Alleles* |
| --- | --- | --- | --- | --- | --- | --- |
| *E* | CotoG12 | TGCAAGTCTTAACTCACCTCATT | CCACTCCCCTAGTTTTCATCTAC | (AC) 23 | 220-256 | 17 |
| *E* | LcM | CTGAGCTGGTTGTGCAGAAG | CTGCATCCCCAGCGCT | (AG) 13 | 188-272 | 29 |
| *E* | LcO | GAGGTCCTGTTTGTGCCAAG | CAGGTCCGCGGTTAATTACG | (AC) 10 | 195-211 | 9 |
| *F* | LcP | ATCTCACATGCTGGGTTAATTT | GCAGGAAAGGGGAGAATCTC | (GTTT) 7 | 244-292 | 5 |
| *F* | LcU | GAAACAGCCTGCATTTACACA | AAATGTCTCCCCTGTCCTCA | (ATCT) 7 | 234-294 | 15 |
| *F* | LbG | CTGGGATCACATGGGGAACT | ATGTGGACTCAGCTCACACA | (AC) 11 | 209-239 | 16 |
| *MP1* | LAS9263AG | GCCTCCCTCGCGCCATCTGTTGGGAGGAGGTGCTG  +6FAM: GCCTCCCTCGCGCCA | GGGAATTATCAGCGGTGCAGG | (AG) 11 | 309 | 2 |
| *MP1* | LAS9284AG | CAGGACCAGGCTACCGTGTGTCACCCTGGACTTGTCCC  +NED: CAGGACCAGGCTACCGTG | GAGGTTTGGTGGCTGATGGG | (AG) 10 | 444 | 4 |
| *MP1* | LAS9367AC | GCCTTGCCAGCCCGCGGCTGACACGTTACTTGGG  +VIC: GCCTTGCCAGCCCGC | TAACACTCCTCTACCCGGCG | (AC) 14 | 236 | 16 |
| *MP2* | LAS6266AG | CGGAGAGCCGAGAGGTGTCTGACACTTGGGTCC  +PET: CGGAGAGCCGAGAGGTG  TGGC | TGGCCCTCATCCCATAATCAC | (AG) 23 | 294 | 16 |
| *MP2* | LAS7831AC | GCCTTGCCAGCCCGCAGGTGGACAGGGAGATTTGAC  +VIC: GCCTTGCCAGCCCGC | TGTCAGCCTGGAAGTACTGC | (AC) 16 | 413 | 17 |
| *MP2* | LAS8539AC | GCCTCCCTCGCGCCACCAGTTCCAGCCTTGCACAG  +6FAM: GCCTCCCTCGCGCCA | TGCTTTGGTGCCTCGAACAG | (AC) 14 | 195 | 10 |
| *MP2* | LAS9555AG | GGAGAGCCGAGAGGTGTGCCTGCATTCCTTCATCCATG  +NED: CGGAGAGCCGAGAGGTG | CCCAAACCCTTGACACTTCC | (AG) 21 | 452 | 19 |
| *MP2* | LAS9618AC | GCCTTGCCAGCCCGCATACCACCTGCTGACCTTGG  +VIC: GCCTTGCCAGCCCGC | GACAGCTGCTCACCCAAATC | (AC) 12 | 178 | 12 |
| *MP5* | LAS4206AC | CGGAGAGCCGAGAGGTGGRGAATTCTGCCTTGACTGGG  +PET: CGGAGAGCCGAGAGGTG | GGACCCAGTGCCAATCCAAC | (AC) 14 | 308 | 3 |
| *MP5* | LAS8830AC | GCCTCCCTCGCGCCAGATGGGAATAAGGACTAGAGTG  +6FAM: GCCTCCCTCGCGCCA | CCAATTTGGCACCATTCCCAG | (AC) 18 | 266 | 10 |
| *MP5* | LAS8843AC | GCCTTGCCAGCCCGCTCAGACAAAGAAGGCCTGTC  +VIC: GCCTTGCCAGCCCGC | CTGAGGACGGGTGYGTTCCC | (AC) 11 | 167 | 8 |
| *MP5* | LAS9141AC | CAGGACCAGGCTACCGTGGTCGCCCAGTGTCCATGATG  +NED: CAGGACCAGGCTACCGTG | CGACCTCCTTGTTCAATACC | (AC) 13 | 307 | 10 |
| *MP5* | LAS9290AC | GCCTTGCCAGCCCGCCAACAGAGAGCTCYATTACC  +VIC: GCCTTGCCAGCCCGC | GRGGCTGTGCTTGTAGTGCC | (AC) 17 | 391 | 16 |
| *MP5* | LAS9524AC | CGGAGAGCCGAGAGGTGCACACAGAAGGCAGCCAATG  +PET: CGGAGAGCCGAGAGGTG | GCACCTGGGCAAGAAGTACG | (AC) 22 | 491 | 13 |
