# Supplementary material for: Genetic diversity, structure, and effective population size of an endangered, endemic hoary bat, ʻōpeʻapeʻa, across the Hawaiian Islands
Source: PeerJ. 2023 Jan 25;11:e14365. doi: 10.7717/peerj.14365 (PMC9884036; doi:10.7717/peerj.14365)
Supplement: Supplemental Information 3 — Number of individuals amplified at locus (n), number of alleles (na), allelic richness (Ar), observed (H o) and expected heterozygosity (He), significance level of Hardy-Weinberg (HWE) tests, null alelle frequencies based on Van Oosterhout et al. (2004), and inbreeding coefficient (FIS). The term mono is used to describe loci that were monomorphic. [file peerj-11-14365-s003.docx]

| Island | Locus | n | Size range (bp) | n_a_ | A_r_ | H_o_ | H_e_ | HWE p-value | Null Allele Frequency | F_IS_ |
| --- | --- | --- | --- | --- | --- | --- | --- | --- | --- | --- |
| Hawai‘i | CotoG12 | 21 | - | 10 | - | 0.571 | 0.828 | < 0.001* | 0.150 | **-** |
|  | LcM | 131 | 199-264 | 24 | 12 | 0.679 | 0.739 | 0.840 | 0.054 | 0.082 |
|  | LcO | 131 | 199-209 | 7 | 3 | 0.213 | 0.236 | 0.420 | 0.049 | 0.096 |
|  | LcP | 131 | 250-257 | 3 | 2 | 0.091 | 0.088 | 1.000 | -0.046 | -0.040 |
|  | LcU | 131 | 259-279 | 6 | 6 | 0.748 | 0.776 | 0.127 | 0.015 | 0.036 |
|  | LbG | 130 | 210-233 | 11 | 7 | 0.761 | 0.794 | 0.526 | 0.017 | 0.041 |
|  | LAS9263AG | 127 | 301-302 | 2 | 1 | 0.007 | 0.007 | 1.000 | -0.003 | < 0.001 |
|  | LAS9284AG | 127 | 424-431 | 5 | 3 | 0.251 | 0.261 | 0.773 | 0.008 | 0.037 |
|  | LAS9367AC | 127 | 218-239 | 10 | 8 | 0.866 | 0.859 | 0.980 | -0.006 | 0.007 |
|  | LAS6266AG | 129 | 309-326 | 10 | 7 | 0.806 | 0.794 | 0.140 | -0.011 | -0.014 |
|  | LAS7831AC | 130 | 423-431 | 5 | 4 | 0.461 | 0.480 | 0.073 | 0.012 | 0.039 |
|  | LAS8539AC | 130 | 197-210 | 8 | 5 | 0.392 | 0.640 | < 0.001* | 0.193 | 0.389 |
|  | LAS9555AG | 129 | 449-508 | 21 | 13 | 0.837 | 0.916 | 0.018 | 0.041 | 0.085 |
|  | LAS9618AC | 130 | 156-179 | 8 | 5 | 0.546 | 0.573 | 0.430 | 0.028 | 0.047 |
|  | LAS4206AC | 131 | 271-286 | 5 | 3 | 0.274 | 0.273 | 0.839 | -0.005 | -0.006 |
|  | LAS8830AC | 130 | 250-276 | 10 | 7 | 0.846 | 0.794 | 0.638 | -0.034 | -0.064 |
|  | LAS8843AC | 131 | 154-164 | 4 | 3 | 0.572 | 0.597 | 0.574 | 0.024 | 0.042 |
|  | LAS9141AC | 131 | 307-322 | 8 | 6 | 0.725 | 0.749 | 0.649 | 0.010 | 0.033 |
|  | LAS9290AC | 131 | 356-392 | 12 | 7 | 0.832 | 0.807 | 0.659 | -0.018 | -0.029 |
|  | LAS9524AC | 130 | 396-424 | 15 | 11 | 0.884 | 0.885 | 0.585 | -0.002 | < -0.001 |
| Maui | CotoG12 | 32 | - | 8 | - | 0.500 | 0.807 | < 0.001* | 0.186 | **-** |
|  | LcM | 98 | 208-256 | 19 | 10 | 0.765 | 0.843 | 0.015 | 0.038 | 0.093 |
|  | LcO | 98 | 201-205 | 4 | 3 | 0.193 | 0.189 | 0.664 | -0.003 | -0.021 |
|  | LcP | 99 | 250-258 | 3 | 3 | 0.353 | 0.317 | 0.036 | -0.094 | -0.115 |
|  | LcU | 99 | 259-279 | 6 | 5 | 0.696 | 0.650 | 0.636 | -0.040 | -0.072 |
|  | LbG | 99 | 217-233 | 9 | 7 | 0.626 | 0.733 | 0.070 | 0.060 | 0.147 |
|  | LAS9263AG | 100 | 301 | 1 | 1 | mono | - | - | 0.000 | - |
|  | LAS9284AG | 100 | 428-431 | 2 | 2 | 0.210 | 0.204 | 1.000 | -0.016 | -0.026 |
|  | LAS9367AC | 101 | 218-236 | 7 | 6 | 0.712 | 0.803 | 0.018 | 0.051 | 0.113 |
|  | LAS6266AG | 98 | 303-330 | 14 | 9 | 0.857 | 0.856 | 0.146 | -0.003 | < -0.001 |
|  | LAS7831AC | 100 | 419-437 | 6 | 4 | 0.380 | 0.392 | 0.269 | 0.009 | 0.031 |
|  | LAS8539AC | 102 | 188-204 | 10 | 6 | 0.509 | 0.706 | < 0.001* | 0.148 | 0.279 |
|  | LAS9555AG | 95 | 450-498 | 19 | 13 | 0.874 | 0.915 | 0.283 | 0.020 | 0.046 |
|  | LAS9618AC | 102 | 156-183 | 10 | 6 | 0.784 | 0.744 | 0.513 | -0.032 | -0.053 |
|  | LAS4206AC | 102 | 273-285 | 3 | 3 | 0.264 | 0.262 | 0.264 | 0.018 | -0.007 |
|  | LAS8830AC | 102 | 250-272 | 6 | 5 | 0.774 | 0.763 | 0.847 | -0.013 | -0.015 |
|  | LAS8843AC | 102 | 153-169 | 6 | 4 | 0.647 | 0.633 | 0.024 | -0.010 | -0.021 |
|  | LAS9141AC | 102 | 310-317 | 6 | 5 | 0.558 | 0.703 | 0.070 | 0.097 | 0.206 |
|  | LAS9290AC | 101 | 367-388 | 6 | 6 | 0.801 | 0.752 | 0.485 | -0.039 | -0.066 |
|  | LAS9524AC | 99 | 388-424 | 14 | 9 | 0.797 | 0.849 | 0.150 | 0.028 | 0.061 |
| O‘ahu | CotoG12 | 34 | - | 7 | - | 0.618 | 0.770 | < 0.001* | 0.051 | - |
|  | LcM | 49 | 214-268 | 11 | 8 | 0.714 | 0.768 | 0.321 | 0.027 | 0.072 |
|  | LcO | 49 | 202-205 | 2 | 2 | 0.122 | 0.116 | 1.000 | -0.063 | -0.055 |
|  | LcP | 47 | 250-258 | 3 | 2 | 0.297 | 0.259 | 0.632 | -0.160 | -0.151 |
|  | LcU | 49 | 263-275 | 5 | 4 | 0.673 | 0.706 | 0.637 | 0.018 | 0.047 |
|  | LbG | 49 | 216-225 | 8 | 7 | 0.734 | 0.801 | 0.029 | 0.035 | 0.084 |
|  | LAS9263AG | 47 | 301 | 1 | 1 | mono | - | - | 0.000 | - |
|  | LAS9284AG | 47 | 431 | 1 | 1 | mono | - | - | 0.000 | - |
|  | LAS9367AC | 47 | 218-236 | 8 | 6 | 0.744 | 0.677 | 0.713 | -0.063 | -0.099 |
|  | LAS6266AG | 47 | 303-330 | 9 | 8 | 0.744 | 0.810 | 0.488 | 0.033 | 0.08 |
|  | LAS7831AC | 48 | 425-433 | 4 | 3 | 0.479 | 0.381 | 0.272 | -0.267 | -0.258 |
|  | LAS8539AC | 48 | 200-201 | 2 | 2 | 0.000 | 0.449 | < 0.001* | 0.393 | 1.000 |
|  | LAS9555AG | 46 | 457-487 | 8 | 7 | 0.760 | 0.771 | 0.271 | 0.003 | 0.013 |
|  | LAS9618AC | 48 | 165-183 | 4 | 4 | 0.729 | 0.622 | 0.670 | -0.057 | -0.101 |
|  | LAS4206AC | 49 | 271-285 | 3 | 2 | 0.061 | 0.060 | 1.000 | -0.030 | -0.014 |
|  | LAS8830AC | 48 | 251-276 | 6 | 5 | 0.833 | 0.744 | 0.822 | -0.066 | -0.120 |
|  | LAS8843AC | 49 | 154-164 | 4 | 3 | 0.428 | 0.427 | 0.414 | 0.003 | -0.003 |
|  | LAS9141AC | 49 | 311-317 | 4 | 4 | 0.755 | 0.656 | 0.056 | -0.112 | -0.152 |
|  | LAS9290AC | 48 | 379-388 | 4 | 4 | 0.666 | 0.625 | 0.426 | -0.019 | -0.006 |
|  | LAS9524AC | 47 | 388-424 | 7 | 6 | 0.382 | 0.538 | < 0.001* | 0.151 | 0.290 |
| Kaua‘i | CotoG12 | - | - | - | - | - | - | - | - | - |
|  | LcM | 16 | 218-268 | 7 | 7 | 0.937 | 0.814 | 0.864 | -0.106 | -0.156 |
|  | LcO | 16 | 203-205 | 2 | 2 | 0.125 | 0.120 | 1.000 | -0.064 | -0.034 |
|  | LcP | 16 | 250 | 1 | 1 | mono | - | - | 0.000 | - |
|  | LcU | 16 | 263-275 | 4 | 4 | 0.500 | 0.719 | 0.246 | 0.135 | 0.312 |
|  | LbG | 16 | 217-225 | 6 | 6 | 0.687 | 0.806 | 0.267 | 0.062 | 0.151 |
|  | LAS9263AG | 16 | 301 | 1 | 1 | mono | - | - | 0.000 | - |
|  | LAS9284AG | 16 | 431 | 1 | 1 | mono | - | - | 0.000 | - |
|  | LAS9367AC | 16 | 218-236 | 6 | 6 | 0.625 | 0.689 | 0.091 | 0.058 | 0.096 |
|  | LAS6266AG | 16 | 312-328 | 6 | 6 | 0.937 | 0.768 | 0.844 | -0.154 | -0.239 |
|  | LAS7831AC | 16 | 425-427 | 2 | 2 | 0.125 | 0.120 | 1.000 | -0.064 | -0.034 |
|  | LAS8539AC | 16 | 200-201 | 2 | 2 | 0.000 | 0.483 | < 0.001* | 0.402 | 1.000 |
|  | LAS9555AG | 16 | 458-499 | 9 | 9 | 0.750 | 0.737 | 0.768 | -0.031 | -0.017 |
|  | LAS9618AC | 16 | 162-173 | 4 | 4 | 0.750 | 0.653 | 0.861 | -0.104 | -0.154 |
|  | LAS4206AC | 16 | 273-285 | 2 | 2 | 0.062 | 0.062 | 1.000 | -0.003 | < 0.001 |
|  | LAS8830AC | 16 | 241-276 | 6 | 6 | 0.812 | 0.848 | 0.800 | 0.003 | 0.044 |
|  | LAS8843AC | 16 | 154-164 | 3 | 3 | 0.375 | 0.491 | 0.099 | 0.085 | 0.243 |
|  | LAS9141AC | 16 | 311-317 | 4 | 4 | 0.625 | 0.610 | 0.680 | -0.059 | -0.023 |
|  | LAS9290AC | 16 | 367-390 | 7 | 7 | 0.687 | 0.675 | 0.741 | -0.008 | -0.018 |
|  | LAS9524AC | 16 | 410-424 | 6 | 6 | 0.937 | 0.850 | 0.145 | -0.072 | -0.105 |

***** significant deviation from HWE after Bonferroni corrections for multiple comparisons.
