# Supplementary material for: Genetic diversity, structure, and effective population size of an endangered, endemic hoary bat, ʻōpeʻapeʻa, across the Hawaiian Islands
Source: PeerJ. 2023 Jan 25;11:e14365. doi: 10.7717/peerj.14365 (PMC9884036; doi:10.7717/peerj.14365)
Supplement: Supplemental Information 4 — Number of sequences obtained (n), number of haplotypes (H), number of polymorphic sites (S), haplotype diversity (Hd) with standard deviation (SD), nucleotide diversity (π), Watterson’s theta estimator (θ) per site and per sequence. [file peerj-11-14365-s004.docx]

| Island | Years | n | H | Number of polymorphic sites (S) | Haplotype diversity (Hd) ± SD | Nucleotide diversity (π) | θ (site/seq) |
| --- | --- | --- | --- | --- | --- | --- | --- |
| Hawai‘i | 2009-2012 | 68 | 8 | 22 | 0.448 ± 0.072 | 0.0015 | 0.007/4.593 |
|  | 2018-2019 | 68 | 8 | 7 | 0.320 ± 0.073 | 0.0006 | 0.002/1.462 |
| Maui | 2012-2014 | 20 | 4 | 24 | 0.668 ± 0.074 | 0.0130 | 0.010/6.765 |
|  | 2016-2017 | 33 | 3 | 23 | 0.561 ± 0.048 | 0.0070 | 0.008/5.667 |
|  | 2018-2019 | 29 | 5 | 24 | 0.717 ± 0.043 | 0.0164 | 0.009/6.111 |
| O‘ahu | 2013 | 13 | 3 | 4 | 0.295 ± 0.156 | 0.0012 | 0.002/1.289 |
|  | 2014 | 10 | 3 | 18 | 0.511 ± 0.164 | 0.0012 | 0.009/6.363 |
|  | 2015-2016 | 10 | 2 | 21 | 0.356 ± 0.159 | 0.0011 | 0.011/7.423 |
|  | 2017-2018 | 9 | 2 | 21 | 0.222 ± 0.166 | 0.0071 | 0.011/7.726 |
| Kaua‘i | 2008-2019 | 16 | 2 | 3 | 0.125 ± 0.106 | 0.0006 | 0.001/0.904 |
