# Supplementary material for: Genetic diversity, structure, and effective population size of an endangered, endemic hoary bat, ʻōpeʻapeʻa, across the Hawaiian Islands
Source: PeerJ. 2023 Jan 25;11:e14365. doi: 10.7717/peerj.14365 (PMC9884036; doi:10.7717/peerj.14365)
Supplement: Supplemental Information 5 — Sample size (n), number of polymorphic loci, allelic richness (Ar), mean number of alleles, mean expected heterozygosity (He), mean observed heterozygosity (Ho), average gene diversity, and population inbreeding coefficient (FIS). Means are reported with standard deviations. [file peerj-11-14365-s005.docx]

| Island | Year | n | Loci | | Ar | | Alleles | | H_e_ | | H_o_ | Gene diversity | | | F_IS_ | |
| --- | --- | --- | --- | --- | --- | --- | --- | --- | --- | --- | --- | --- | --- | --- | --- | --- |
| Hawai‘i | 2009-2010 | 33 | 14 | 5.12 | | 7.05 ± 4.07 | | 0.62 ± 0.25 | | 0.61 ± 0.27 | | | 0.61 ± 0.31 | 0.002 | |  |
|  | 2011-2013 | 18 | 17 | 5.38 | | 6.16 ± 3.76 | | 0.57 ± 0.31 | | 0.55 ± 0.31 | | | 0.57 ± 0.29 | 0.024 | |  |
|  | 2014-2017 | 12 | 17 | 5.28 | | 5.52 ± 2.91 | | 0.61 ± 0.29 | | 0.62 ± 0.26 | | | 0.58 ± 0.31 | 0.024 | |  |
|  | 2018 | 17 | 14 | 5.31 | | 6.75 ± 3.62 | | 0.65 ± 0.25 | | 0.62 ± 0.25 | | | 0.62 ± 0.32 | 0.049 | |  |
|  | 2019 | 52 | 17 | 5.33 | | 8.47 ± 4.55 | | 0.63 ± 0.25 | | 0.63 ± 0.25 | | | 0.59 ± 0.30 | -0.001 | |  |
| Maui | 1988-1999 | 4 | 10 | - | | 2.62 ± 1.03 | | 0.63 ± 0.24 | | 0.63 ± 0.28 | | | 0.44 ± 0.28 | -0.-0.120 | |  |
|  | 2009-2011 | 9 | 17 | 4.61 | | 4.82 ± 2.21 | | 0.62 ± 0.26 | | 0.53 ± 0.27 | | | 0.57 ± 0.31 | 0.135 | |  |
|  | 2012-2013 | 12 | 17 | 4.99 | | 5.76 ± 3.44 | | 0.64 ± 0.26 | | 0.57 ± 0.25 | | | 0.60 ± 0.32 | 0.102 | |  |
|  | 2014-2015 | 11 | 17 | 4.66 | | 5.17 ± 2.29 | | 0.65 ± 0.22 | | 0.68 ± 0.27 | | | 0.62 ± 0.32 | -0.040 | |  |
|  | 2016 | 13 | 16 | 4.63 | | 5.29 ± 2.78 | | 0.63 ± 0.27 | | 0.65 ± 0.27 | | | 0.57 ± 0.30 | -0.049 | |  |
|  | 2017 | 23 | 12 | 4.40 | | 5.82 ± 3.48 | | 0.61 ± 0.25 | | 0.59 ± 0.23 | | | 0.58 ± 0.30 | -0.173 | |  |
|  | 2018 | 18 | 12 | 4.48 | | 5.58 ± 2.87 | | 0.59 ± 0.26 | | 0.63 ± 0.30 | | | 0.53 ± 0.28 | -0.075 | |  |
|  | 2019-2020 | 12 | 17 | 4.63 | | 5.35 ± 2.78 | | 0.59 ± 0.24 | | 0.55 ±0.22 | | | 0.56 ± 0.29 | 0.096 | |  |
| O‘ahu | 2011-2012 | 5 | 14 | 2.72 | | 3.21 ± 1.05 | | 0.54 ± 0.23 | | 0.61 ± 0.31 | | | 0.42 ± 0.24 | -0.151 | |  |
|  | 2013 | 13 | 7 | 3.01 | | 4.31 ± 1.70 | | 0.51 ± 0.25 | | 0.54 ± 0.27 | | | 0.48 ± 0.28 | -0.097 | |  |
|  | 2014 | 10 | 12 | 3.30 | | 4.25 ± 1.88 | | 0.58 ± 0.27 | | 0.58 ± 0.31 | | | 0.54 ± 0.28 | -0.039 | |  |
|  | 2015 – 2016 | 10 | 15 | 3.21 | | 4.67 ± 1.79 | | 0.63 ± 0.19 | | 0.63 ± 0.18 | | | 0.55 ± 0.30 | -0.035 | |  |
|  | 2017 -2018 | 10 | 15 | 2.98 | | 4.00 ± 1.41 | | 0.61 ± 0.17 | | 0.65 ± 0.21 | | | 0.50 ± 0.27 | -0.070 | |  |
