# Supplementary material for: Genetic diversity, structure, and effective population size of an endangered, endemic hoary bat, ʻōpeʻapeʻa, across the Hawaiian Islands
Source: PeerJ. 2023 Jan 25;11:e14365. doi: 10.7717/peerj.14365 (PMC9884036; doi:10.7717/peerj.14365)
Supplement: Supplemental Information 6 — Pairwise FST values for microsatellites in ʻōpeʻapeʻa (Hawaiian hoary bat: Lasiurus semotus) adjusted for null alleles using the ENA method in FreeNA (Chaupis & Estoup, 2007) are located below the diagonal, while associated 95% confidence intervals are given in brackets above the diagonal. [file peerj-11-14365-s006.docx]

|  | Island | Hawai‘i | Maui | O‘ahu | Kaua‘i |
| --- | --- | --- | --- | --- | --- |
| Microsatellite | Hawai‘i | - | [0.030-0.065] | [0.061-0.126] | [0.065-0.124] |
| Adjusted using ENA method | Maui | 0.046 | - | [0.057-0.134] | [0.059-0.115] |
|  | O‘ahu | 0.093 | 0.093 | - | [0.029-0.075] |
|  | Kaua‘i | 0.096 | 0.085 | 0.052 | **-** |
