# Supplementary material for: Genetic diversity, structure, and effective population size of an endangered, endemic hoary bat, ʻōpeʻapeʻa, across the Hawaiian Islands
Source: PeerJ. 2023 Jan 25;11:e14365. doi: 10.7717/peerj.14365 (PMC9884036; doi:10.7717/peerj.14365)
Supplement: Supplemental Information 7 — Additional estimates of contemporary effective population size (NeC) by collection year period based on microsatellite linkage disequilibrium and 95% confidence intervals (CIs) from jackknife and parametric resampling for ʻōpeʻapeʻa (Hawaiian hoary bat: Lasiurus semotus). Values were computed with Ne Estimator V2 (Do et al., 2014). [file peerj-11-14365-s007.docx]

| Island | Years | n | N_eC_ | Jackknife 95% CI | Parametric 95% CI |
| --- | --- | --- | --- | --- | --- |
| Hawai‘i | 2009-2012 | 45 | 391 | 125 - ∞ | 138 – ∞ |
|  | 2018-2019 | 70 | 350 | 115 – ∞ | 174 – 4,262 |
| Maui | 2012-2014 | 20 | 65 | 33 - 401 | 33 - 364 |
|  | 2016-2019 | 64 | 95 | 47 -436 | 64 - 161 |
| O‘ahu | 2013-2015 | 29 | 21 | 13 - 42 | 15 - 31 |
|  | 2017-2020 | 11 | 11 | 4 - 35 | 5 - 27 |
| Kaua‘i | 2008-2019 | 16 | 91 | 17 - ∞ | 29 - ∞ |
