# Supplementary material for: Genetic diversity, structure, and effective population size of an endangered, endemic hoary bat, ʻōpeʻapeʻa, across the Hawaiian Islands
Source: PeerJ. 2023 Jan 25;11:e14365. doi: 10.7717/peerj.14365 (PMC9884036; doi:10.7717/peerj.14365)
Supplement: Supplemental Information 8 — Fu’s Fs values for ʻōpeʻapeʻa (Hawaiian hoary bat: Lasiurus semotus) by island rarified to the number of cytochrome c oxidase I (COI) mitochondrial sequences available for each island. [file peerj-11-14365-s008.docx]

| Number of Sequences | Hawai‘i | Maui | O‘ahu | Kaua‘i |
| --- | --- | --- | --- | --- |
| 16 | 2.066 | 8.633 | 8.90 | 0.769 |
| 47 | -6.92 | 12.19 | 1.745 | - |
| 92 | -9.21 | 12.46 | - | - |
| 166 | -14.48 | - | - | - |
