# Supplementary material for: Genetic diversity, structure, and effective population size of an endangered, endemic hoary bat, ʻōpeʻapeʻa, across the Hawaiian Islands
Source: PeerJ. 2023 Jan 25;11:e14365. doi: 10.7717/peerj.14365 (PMC9884036; doi:10.7717/peerj.14365)
Supplement: Supplemental Information 9 — Tajima’s D and Fu’s Fs values for ʻōpeʻapeʻa (Hawaiian hoary bat: Lasiurus semotus) from cytochrome c oxidase I (COI) mitochondrial sequences by island and collection year period. ** P < 0.001, * P < 0.001 [file peerj-11-14365-s009.docx]

| Island | Years | n | Tajima’s D | Fu’s F_s_ |
| --- | --- | --- | --- | --- |
| Hawai‘i | 2009-2012 | 68 | -2.44** | -2.459 |
|  | 2018-2019 | 68 | -2.00* | -7.289 |
| Maui | 2012-2014 | 20 | 1.02 | 9.18 |
|  | 2016-2017 | 33 | -0.64 | 9.25 |
|  | 2018-2019 | 29 | 2.74** | 11.76 |
| O‘ahu | 2013 | 13 | -1.44 | 0.185 |
|  | 2014 | 10 | 1.33 | 7.041 |
|  | 2015-2016 | 10 | 0.02 | 9.705 |
|  | 2017-2018 | 9 | -1.95** | 6.854 |
| Kaua‘i | 2008-2019 | 16 | -1.69 | 0.769 |

** P < 0.001, * P < 0.001
