# Supplementary figures and images for: Genetic diversity, structure, and effective population size of an endangered, endemic hoary bat, ʻōpeʻapeʻa, across the Hawaiian Islands
Source: PeerJ. 2023 Jan 25;11:e14365. doi: 10.7717/peerj.14365 (PMC9884036; doi:10.7717/peerj.14365)

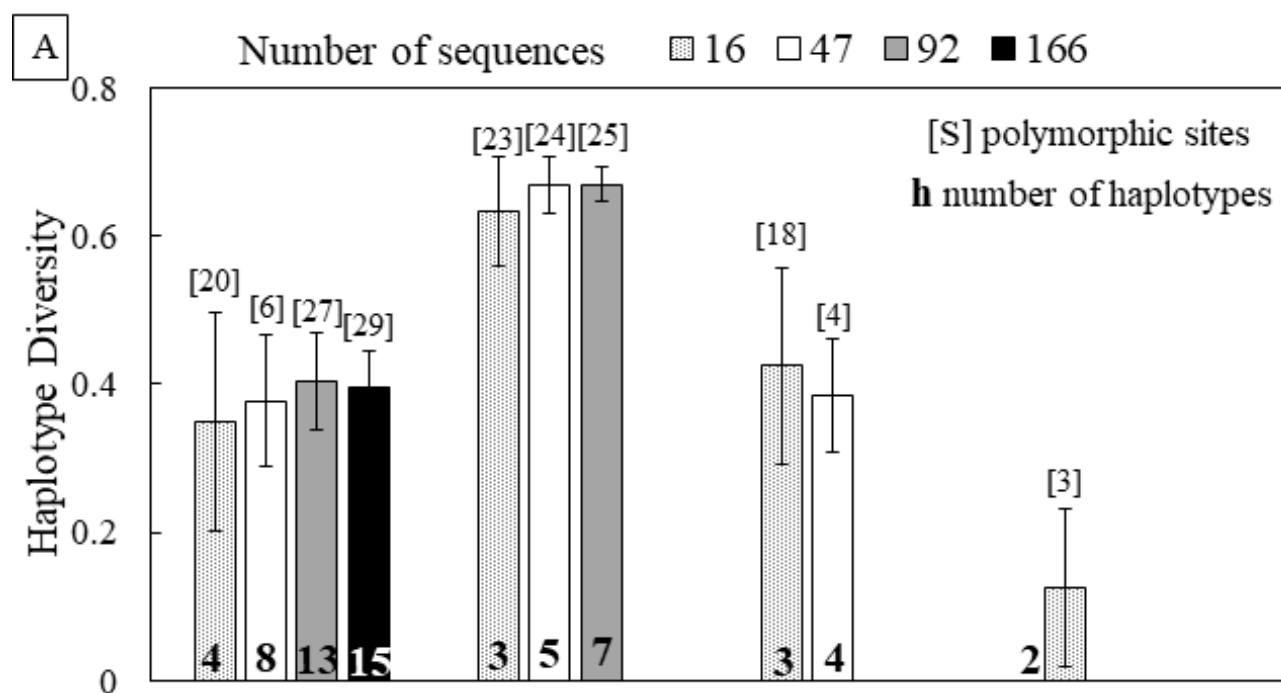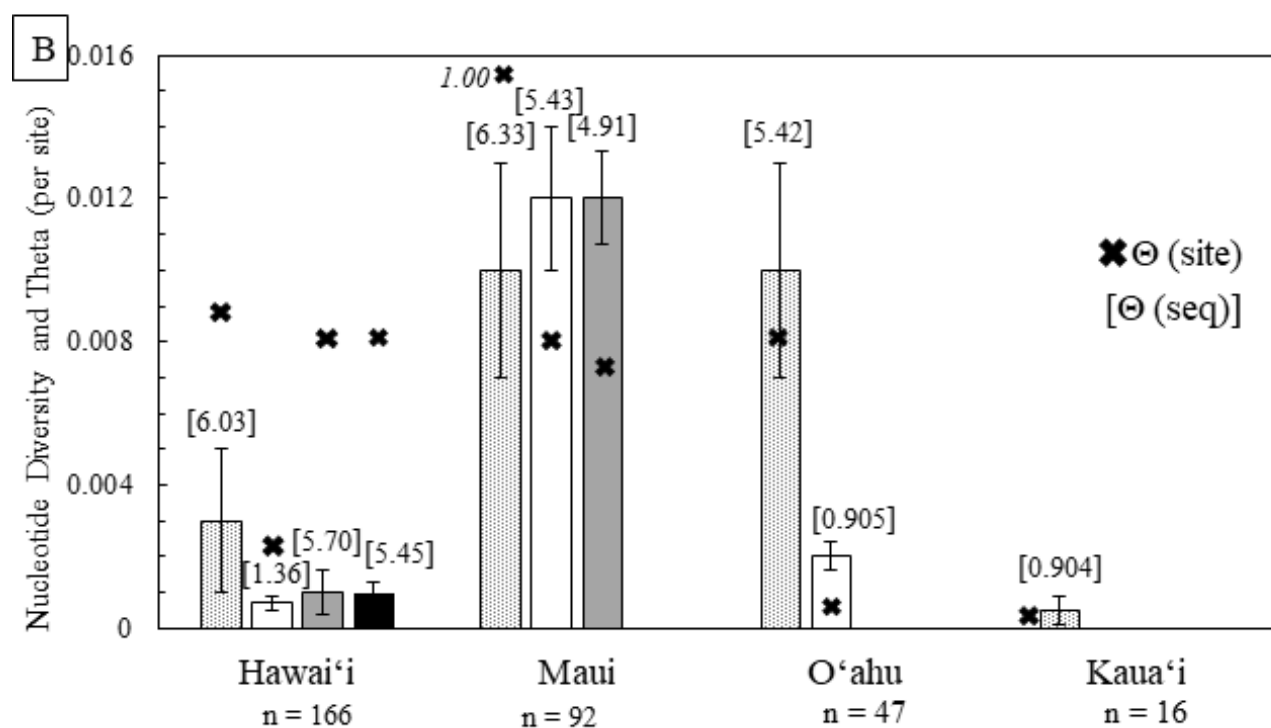

Supplement: Supplemental Information 10 — (A) number of haplotypes (h), number of polymorphic sites (S) appear above bars in brackets, mean haplotype diversity (Hd). (B) mean nucleotide diversity (π), Watterson’s theta per site (bold x), and Watterson’s theta per sequence in brackets above bars. Means are reported error bars that represent standard deviations. [file peerj-11-14365-s010.pdf]

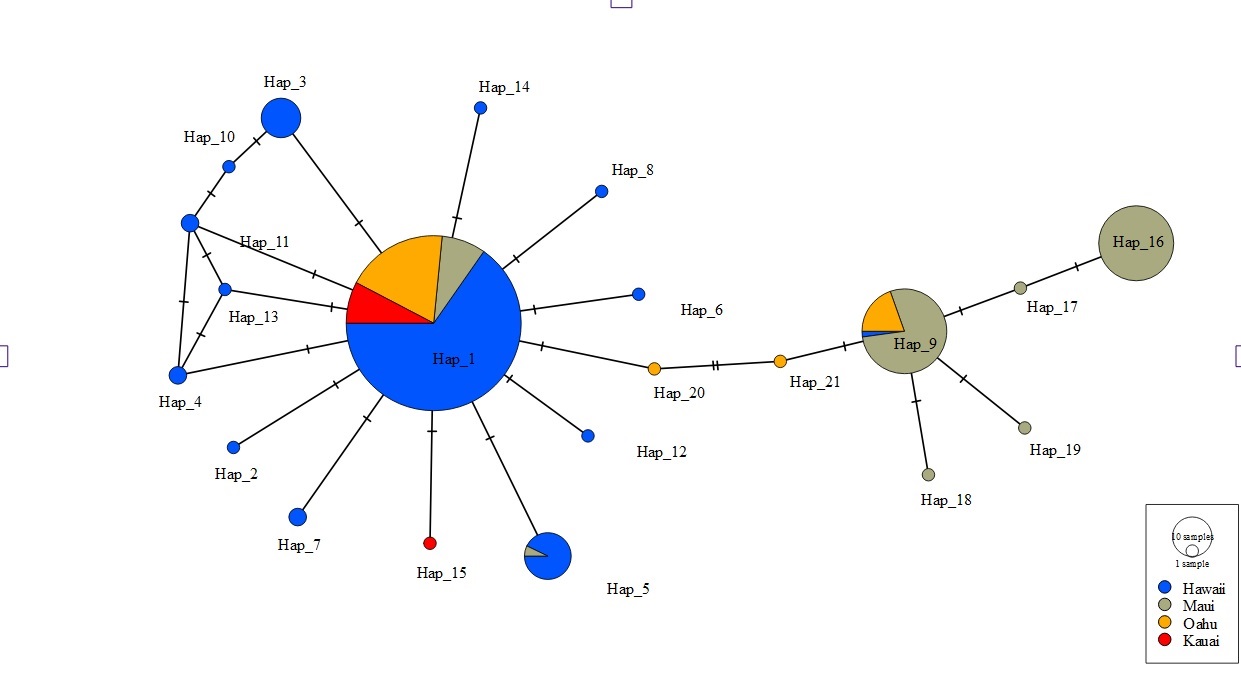

Supplement: Supplemental Information 12 — Haplotype parsimony network constructed from 21 unique mitochondrial haplotypes of the CO1 region of 321 ʻōpeʻapeʻa (Hawaiian hoary bat: Lasiurus semotus), including 5 individuals from Oʻahu with potential heteroplasmy. Unique haplotypes are represented by a colored circle, while islands are defined by separate colors. Lines with dashes between circles represent number of base pair changes between two haplotypes. Number of samples in each haplotype correspond to circle sizes in legend. [file peerj-11-14365-s012.jpg]
