# Supplementary material for: Genetic diversity, structure, and effective population size of an endangered, endemic hoary bat, ʻōpeʻapeʻa, across the Hawaiian Islands
Source: PeerJ. 2023 Jan 25;11:e14365. doi: 10.7717/peerj.14365 (PMC9884036; doi:10.7717/peerj.14365)
Supplement: Supplemental Information 11 — Pie charts represent the haplotype frequencies detected at each collection site. Size of the circle reflects the number of individuals from that collection site, larger circles indicate more individuals collected. [file peerj-11-14365-s011.pdf]

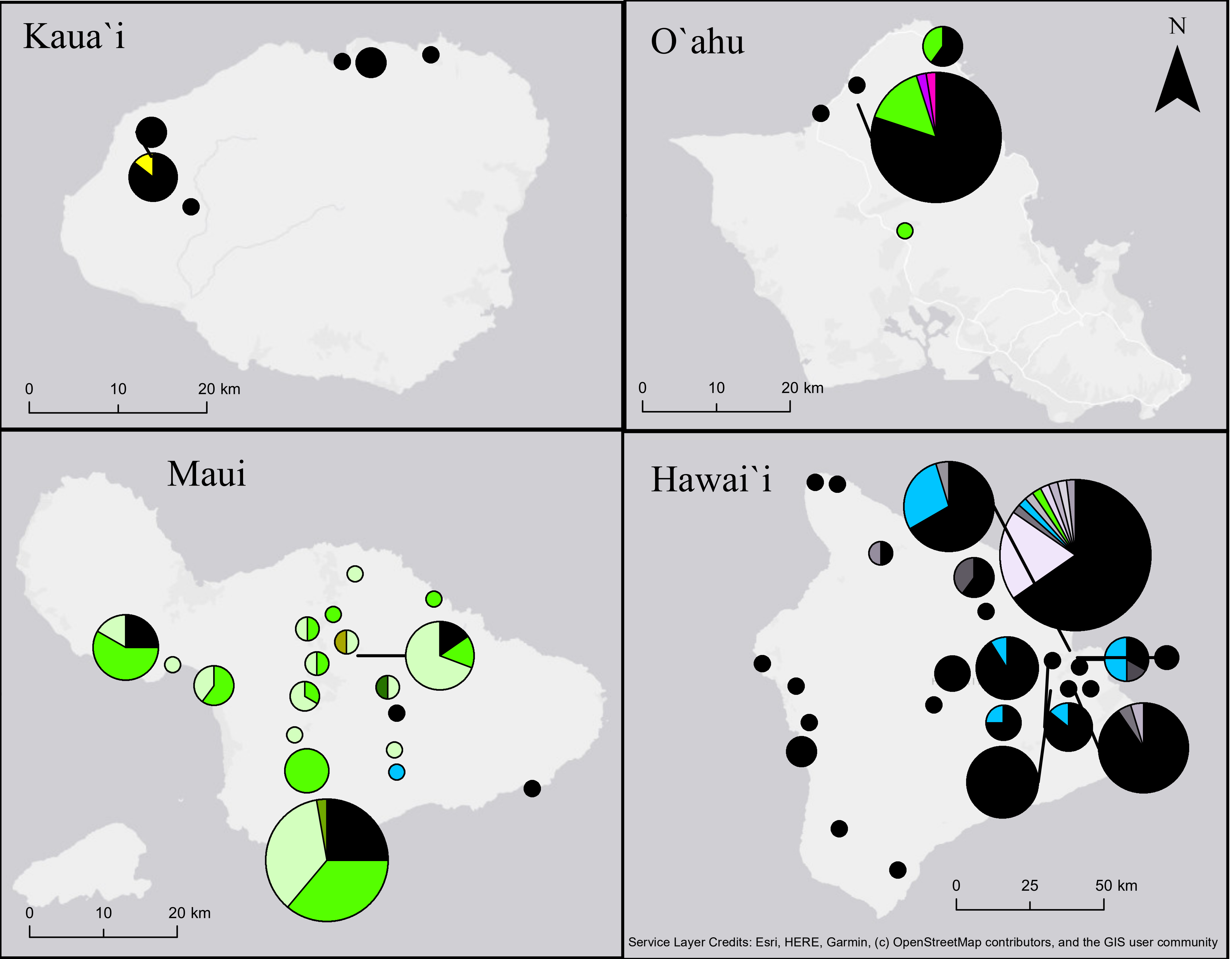

Kaua`i
0
10
20 km
O`ahu
N
0
10
20 km
Maui
0
10
20 km
Hawai`i
0
25
50 km
Service Layer Credits: Esri, HERE, Garmin, (c) OpenStreetMap contributors, and the GIS user community
