# Supplementary material for: Genetic diversity, structure, and effective population size of an endangered, endemic hoary bat, ʻōpeʻapeʻa, across the Hawaiian Islands
Source: PeerJ. 2023 Jan 25;11:e14365. doi: 10.7717/peerj.14365 (PMC9884036; doi:10.7717/peerj.14365)
Supplement: Supplemental Information 13 — Distribution of mitochondrial clade and potential identification of migrant ancestry for individual ʻōpeʻapeʻa (Hawaiian hoary bat: Lasiurus semotus) over ancestry membership in program Structure (Pritchard, Stephens & Donnelly, 2000) plots for Hawaiʻi, Maui, Oʻahu, and Kauaʻi. [file peerj-11-14365-s013.pdf]

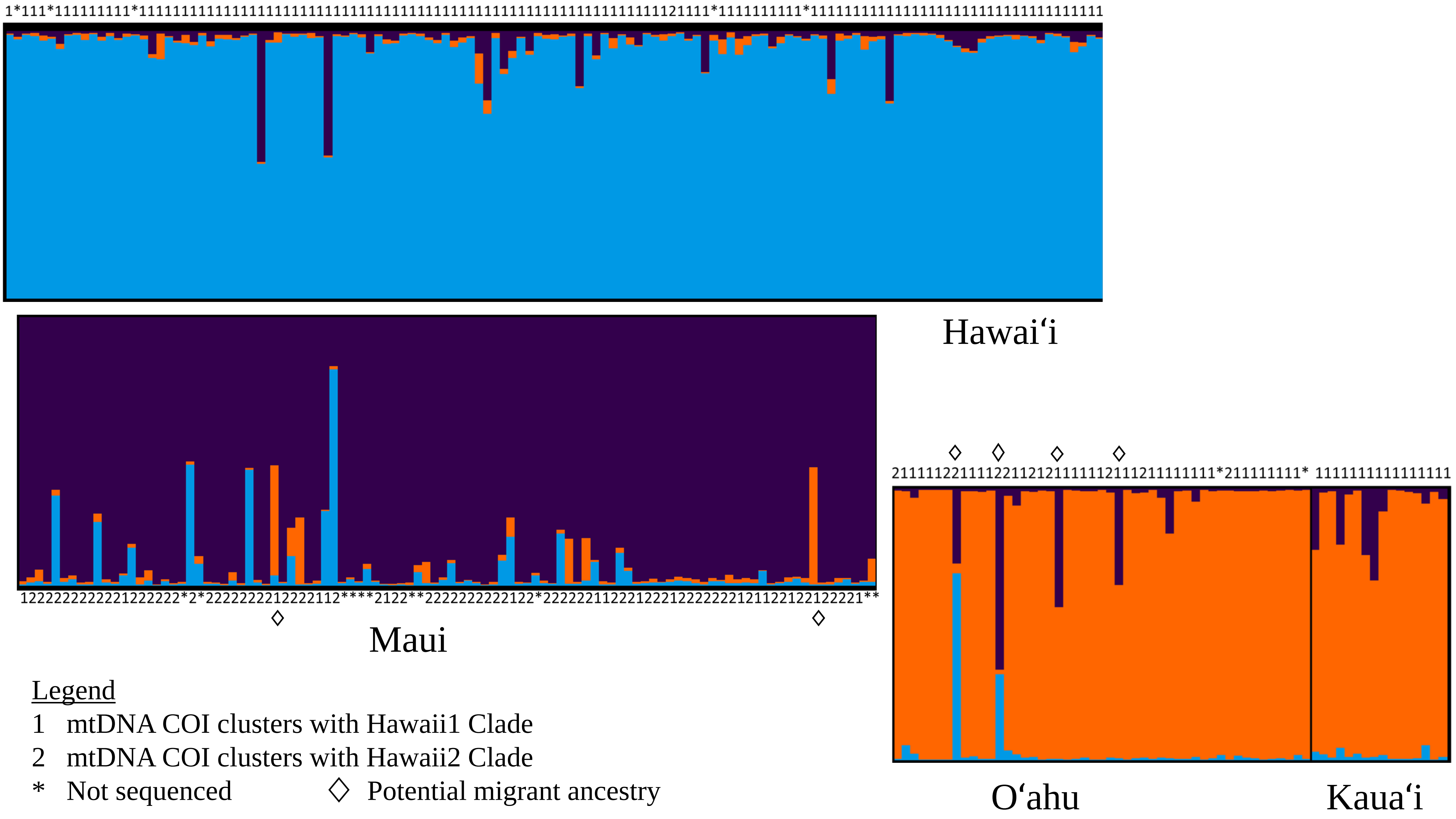
Hawaiʻi
Maui
Oʻahu
Kauaʻi
Legend
1 mtDNA COI clusters with Hawaii1 Clade
2 mtDNA COI clusters with Hawaii2 Clade
* Not sequenced
◊ Potential migrant ancestry
